# Challenges and benefits of integrating diverse sampling strategies in the observation of cardiovascular risk factors (ORISCAV-LUX 2) study

**DOI:** 10.1186/s12874-019-0669-0

**Published:** 2019-02-04

**Authors:** Ala’a Alkerwi, Jessica Pastore, Nicolas Sauvageot, Gwenaëlle Le Coroller, Valéry Bocquet, Marylène d’Incau, Gloria Aguayo, Brice Appenzeller, Dritan Bejko, Torsten Bohn, Laurent Malisoux, Sophie Couffignal, Stephanie Noppe, Charles Delagardelle, Jean Beissel, Anna Chioti, Saverio Stranges, Jean-Claude Schmit

**Affiliations:** 1 Luxembourg Institute of Health (LIH), Department of Population Health, 1A rue Thomas Edison, L-1445 Strassen, Luxembourg; 2 Centre Hospitalier du Luxembourg (CHL), Luxembourg City, Luxembourg; 3 Ministry of Health, Directorate of Health, Luxembourg City, Luxembourg; 4 Department of Epidemiology & Biostatistics, Schulich School of Medicine & Dentistry, Western University, London, ON N6A 5C1 Canada

**Keywords:** Sample representativeness, Population-based study, Follow-up studies, population health, Epidemiology

## Abstract

**Background:**

It is challenging to manage data collection as planned and creation of opportunities to adapt during the course of enrolment may be needed. This paper aims to summarize the different sampling strategies adopted in the second wave of Observation of Cardiovascular Risk Factors (ORISCAV-LUX, 2016–17), with a focus on population coverage and sample representativeness.

**Methods:**

Data from the first nationwide cross-sectional, population-based ORISCAV-LUX survey, 2007–08 and from the newly complementary sample recruited via different pathways, nine years later were analysed. First, we compare the socio-demographic characteristics and health profiles between baseline participants and non-participants to the second wave. Then, we describe the distribution of subjects across different strategy-specific samples and performed a comparison of the overall ORISCAV-LUX2 sample to the national population according to stratification criteria.

**Results:**

For the baseline sample (1209 subjects), the participants (660) were younger than the non-participants (549), with a significant difference in average ages (44 vs 45.8 years; *P* = 0.019). There was a significant difference in terms of education level (*P* < 0.0001), 218 (33%) participants having university qualification vs. 95 (18%) non-participants. The participants seemed having better health perception (*p* < 0.0001); 455 (70.3%) self-reported good or very good health perception compared to 312 (58.2%) non-participants. The prevalence of obesity (*P* < 0.0001), hypertension (*P* < 0.0001), diabetes (*P* = 0.007), and mean values of related biomarkers were significantly higher among the non-participants. The overall sample (1558 participants) was mainly composed of randomly selected subjects, including 660 from the baseline sample and 455 from other health examination survey sample and 269 from civil registry sample (constituting in total 88.8%), against only 174 volunteers (11.2%), with significantly different characteristics and health status. The ORISCAV-LUX2 sample was representative of national population for geographical district, but not for sex and age; the younger (25–34 years) and older (65–79 years) being underrepresented, whereas middle-aged adults being over-represented, with significant sex-specific difference (*p* < 0.0001).

**Conclusion:**

This study represents a careful first-stage analysis of the ORISCAV-LUX2 sample, based on available information on participants and non-participants. The ORISCAV-LUX datasets represents a relevant tool for epidemiological research and a basis for health monitoring and evidence-based prevention of cardiometabolic risk in Luxembourg.

**Electronic supplementary material:**

The online version of this article (10.1186/s12874-019-0669-0) contains supplementary material, which is available to authorized users.

## Background

The optimal allocation of available resources is the concern of every investigator and decision-maker before choosing a population-based study design [1]. Despite the well-known benefits of conducting longitudinal surveys to advance epidemiology and clinical research, full baseline sample participation in follow-up studies is challenging. Over time, initial participants may drop out of the study due to death, move abroad or simply refuse to respond to the successive rounds of surveys, due to loss of interest for added complex examinations and time consuming measurements. This poor compliance and low participation rate may impact dataset quality and sample relevance.

The “Observation of Cardiovascular Risk Factors in Luxembourg” (ORISCAV-LUX) survey, conducted between November 2007 and January 2009, was the first nationwide cross-sectional survey of cardiovascular health monitoring in Luxembourg [2]. It aimed to establish baseline information on the prevalence of “traditional” cardiovascular risk factors, including obesity, hypertension, diabetes mellitus, lipid disorder, smoking and physical inactivity among the general adult population. Complete details about study design, sampling scheme, non-response handling, sample representativeness of the population were published elsewhere [2, 3]. Briefly, a total of 1432 subjects (response rate 32.2%) were successfully recruited, slightly beyond the estimated necessary sample size and the expected participation rate. The comparison of participants and non-participants in the ORISCAV-LUX survey revealed that their distribution and profiles were comparable in terms of cardiovascular morbidity indicators, including prescribed medications, hospital admission and medical measures [3].

From a public health and research perspective, the health surveys need to be repeated at regular intervals to monitor the evolution and allow the development of coherent and effective strategies of prevention. In 2016, the second wave ORISCAV-LUX study was initiated to follow-up the same baseline sample of participants. An extended set of health indicators, new clinical examinations and self-reported information were integrated in the second round of data collection.

Indeed, reaching a suitable number of participants, based on the initial baseline ORISCAV-LUX sample, was challenging. A nationally representative sample is a prerequisite to meet public health goals. In this respect, we had to adapt our planning and suggest alternative solutions to our sampling scheme in order to ensure sufficient sample size, and hence the validity of constituted dataset and the resulting statistics. The objective of this paper is to summarize the different sampling strategies adopted in the ORISCAV-LUX2, with a focus on the evaluation of population coverage and the sample representativeness. Operational issues associated with the implementation of this adaptive sampling schemes were described hereafter in the methodology.

## Methods

### Data collection procedures

Similar to the ORISCAV-LUX baseline study [2], the participation to the second wave included 3 main steps: filling in a self-reported questionnaire; clinical and anthropometric measurements according to standardised operating procedures; and blood, urine and hair samples collection.

The participants in the baseline study received an invitation letter together with an information leaflet, a coupon-answer and a pre-paid envelop, suggesting them to take part in the second wave. The subjects who accepted to participate were asked either to fill in the online questionnaire accessible with a unique identification code, or simply request a paper version indicating their preferred language (French, German, Portuguese or English). The consented subjects were rapidly contacted by phone, to schedule an appointment at one of the nearest study centres.

### Added questionnaires

Several new questionnaires were added, including a self-administered questionnaire filled by the participant at home and another one focusing on the medical aspects completed during the interview by the research nurse. Information on demographic and socio-economic characteristics, personal and family history as well as lifestyle-related questionnaires were collected based on the same tools as the baseline study. New general health status modules were introduced including quality of life 36-Item Short Form Health Survey (SF-36) [4], evaluation of autonomy [Activities of Daily Living (ADL) and Instrumental Activities of Daily Living (IADL) instruments] [5], sleep habits [6], Mini-Mental State Examination test [7], [Centre for Epidemiologic Studies Depression Scale (CES-D)] [8], constipation [9], social support, women’s health, cardiovascular history, detailed personal diseases and chronic conditions, medication, vitamins and supplements intake and pollution-related questionnaire (Please see Additional files 1 and 2). An electronic version of a 174-item Food Frequency Questionnaire (e-FFQ) was also used in the second wave.

### New anthropometric and clinical examinations

In addition to weight, height, waist, and hip circumferences, proximal thigh girth and bio-impedancemetry body composition (Tanita® BC 418) were measured. Further parameters concerning cardiac function including triple blood pressure and pulse rate measurements in sitting and supine position, ECG, pulse wave velocity (Complior®); physical function (including finger tapping, grip strength, balance, chair rises, walking speed, and step test by using Actiheart® were also incorporated. Objective measures of physical activity (7-day accelerometer data by using Actigraph® accelerometer), as well as mental function (five cognitive tests by using the Cambridge Neuropsychological Test Automated Battery CANTAB®) were also collected.

### Sampling schemes

#### Original baseline sample enrolment

In December 2015, the baseline 1432 participants were re-contacted to take part in the second round, except those who had already refused (15 subjects) to take part in follow-up studies. During the 9 years, the missions of the Inspectorate of Social Security (IGSS) who provided the initial sample based on the National Insurance Registry were reformed. This institution was no longer allowed to share nominative data and therefore unable to update the addresses of the participants. They could however confirmed the crude numbers of subjects who quitted the country to live abroad (51) and deceased (23), without link to the identification code, yielding a total eligible sample of 1343 addresses. To avoid sending useless invitations to inexistent subjects, an active research on national website www.editus.lu, as well as direct phone calls were performed to confirm the accuracy of delivery addresses and to correct potential changes.

**Fig. 1:**
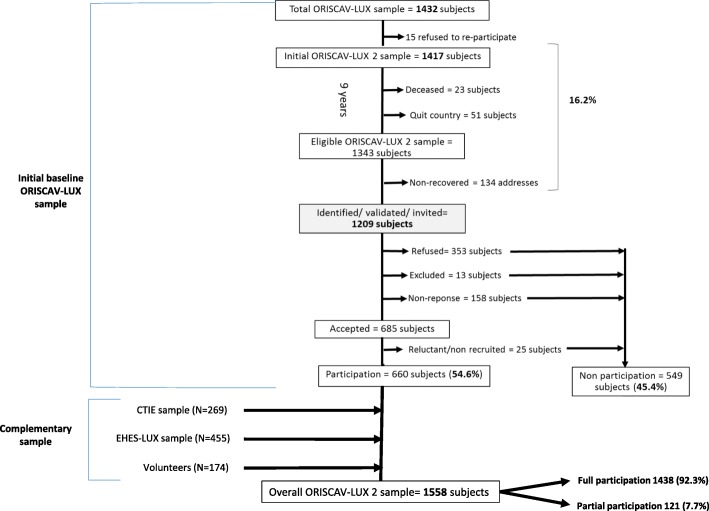
The overall sample participating in the second wave from the different sampling procedures

Following this procedure, further 134 addresses (10% of the eligible sample) could not be found and hence were categorised as “non-recovered”. Then, the invitations were sent to the final identified and validated sample of 1209 addresses. Out of these, 353 (29.2%) refused to take part in the second wave, 158 (13.1%) never answered after three reminders. Further 13 (1.1%) were excluded during the recruitment process due to their move abroad, physical disability or language incapacity. After this scheme, a total of 685 subjects accepted to participate. Among them, 25 subjects (3.6%) did not attend, or cancelled their repeated appointments, and could not be enrolled until the end of the study and hence were categorised as “reluctant/non recruited”. Finally, 660 subjects, constituting 54.6% of the invited sample (Fig. 1).

#### Alternative strategies

To overcome the drop of the initial sample size and preserve a nationally representative sample, three alternative sampling strategies were thereafter implemented to recruiting a new complementary sample from:

1) The civil national registry: With the support of the Ministry of Health and in collaboration with the Government IT Centre [*Centre des Technologies de l’Information de l’Etat* (CTIE)], a new additional random sample of 4737 subjects, accounting for a large anticipated non-participation rate was selected. This number was calculated based on the initial sampling procedure used in the first wave [3]. According to its legal status, the CTIE is the sole institution possessing the nominative information about all resident people in Luxembourg and is apt to approach directly the residents via a nominative mailing. In this context, short letters were sent to the selected subjects summarizing briefly the objective of the study and asking them to send their complete address to the recruiting institute [Luxembourg institute of Health (LIH)] in case of consent. Once the invited subjects agreed to send their personal data via the email dedicated to the project or via a phone call, they were registered in our databank. Thereafter, the same enrolment process begun by sending detailed information about the study and the consented subjects were contacted by our administrative assistant to fix an appointment in our premises. For logistic and practical considerations, the CTIE mailing was dispatched in several batches, each sent to almost 500 subjects, over a period of 6 months. Despite the huge efforts to prepare and organise this procedure, it seemed unhelpful; participation rate constituted only 5.7% (269 participants out of almost 4700 invited subjects).

2) European Health Examination Survey (EHES-LUX) list of participants: Using an existing address list of participants who took part in EHES-LUX study carried out by the LIH. Out of a total of 1431 subjects invited, 455 participants were recruited for the ORISCAV-LUX 2, constituting a participation rate of almost 32%.

3) Volunteers: A call for volunteers was advertised through divers means of communication, for example the LIH social networks (Facebook, Twitter), ORISCAV-LUX project’s website (www.oriscav.lih.lu), the national press, the media, and during outreach events for the general public. For this purpose, study-oriented poster and leaflet were prepared in order to attract new participants. Through this pathway, further 174 volunteers were enrolled (Fig. 1).

Between January 2016 and January 2018, a total of 1558 subjects were recruited in the second wave of the study, including 1438 participants (92.3%) with full participation, and 121 (7.7%) with partial participation. Full participation means that the participants filled in the self-reported questionnaires, attended their appointments and underwent clinical and anthropometric examinations, and provided blood urine and samples. Partial participation entails that the participants answered only self-reported questionnaires, without attending the nurse interview in our study centres.

### Statistical methods

Using the baseline ORISCAV-LUX sample, the socio-demographic characteristics and health profiles between participants and non-participants to the second wave were compared. Then, the distribution of subjects across different strategies of sampling was described. A comparison of the overall ORISCAV-LUX 2 sample to the national population according to stratification criteria (age, sex and geographical district) was performed.

Results were presented as numbers (percentages) for categorical variables and mean ± standard deviation (SD) for continuous variables, by using chi-squared test and one-way ANOVA, respectively. All statistical analyses were performed with Predictive Analytics Software “PASW for Window® version 21.0 software (formerly SPSS Statistics Inc., Chicago, IL, USA)”; *p* < 0.05 was considered statistically significant.

## Results

Based on baseline sample (1209 subjects), Table 1 compares the demographic, socio-economic and cardiometabolic risk profiles of participants and non-participants in ORISCAV-LUX2 study. The participants were significantly younger, with no sex-specific difference. There was a significant difference in terms of education level (*P* < 0.0001), 218 participants having university qualification (33%) vs. 95 non-participants (18%). The participants seemed having a better health perception (*p* < 0.0001); 455 (70.3%) self-reported good or very good health perception Compared to 312 (58.2%) non-participants.

**Table 1:** Comparison of participants versus non-participants based on the baseline ORISCAV-LUX sample (1209 subjects)

Subjects’ characteristics	Participants	Non-participants	*p* value
	*N = 660*	*N = 549*	
Age, year	44.02 ± 11.9	45.79 ± 13.9	0.019
Sex, Men %	335 (50.8%)	255 (46.4%)	0.13
District, %			0.38
Luxembourg	465 (70.6%)	401 (73.0%)	
Diekirch	102 (15.5%)	86 (15.7%)	
Grevenmacher	92 (14.0%)	62 (11.3%)	
Education level,%			< 0.0001
No diploma	110 (16.8%)	188 (34.6%)	
Secondary level	326 (49.8%)	261 (48.0%)	
University level	218 (33.3%)	95 (17.5%)	
Marital status, %			0.08
Married	490 (74.4%)	374 (68.1%)	
Single	105 (15.9%)	107 (19.5%)	
Divorced	52 (7.9%)	50 (9.1%)	
Widowed	12 (1.8%)	18 (3.3%)	
Country of birth,%			0.01
Luxembourg	408 (61.9%)	350 (63.8%)	
Portugal	64 (9.7%)	78 (14.2%)	
Other European country	152 (23.1%)	93 (16.9%)	
Non-European country	35 (5.3%)	28 (5.1%)	
Physical activity, %			0.3
Inactive	101 (16.0%)	101 (19.4%)	
Moderately active	179 (28.3%)	147 (28.2%)	
Active	352 (55.7%)	273 (52.4%)	
Self-reported health perception, %			< 0.0001
Very good	70 (10.8%)	37 (6.9%)	
Good	385 (59.5%)	275 (51.3%)	
Average	169 (26.1%)	204 (38.1%)	
Bad	19 (2.9%)	18 (3.4%)	
Very bad	4 (0.6%)	2 (0.4%)	
Cardiometabolic risk profile			
Smokers,%	113 (17.1%)	120 (21.9%)	0.04
Diabetes, %	19 (2.9%)	33 (6.2%)	0.007
Serum glucose, mg/ml	93.4 ± 14.3	96.6 ± 20.5	0.01
Hypertension,%	234 (35.5%)	255 (46.5%)	< 0.0001
Blood pressure, mmHg			
SBP	128.4 ± 16.0	132.3 ± 18.8	0.001
DBP	82.2 ± 10.8	83.4 ± 11.2	0.04
BMI, kg/m2	25.9 ± 4.6	27.4 ± 5.2	< 0.0001
Obesity, %	122 (18.5%)	155 (28.3%)	< 0.0001
Serum cholesterol, mg/dl	122.5 ± 32.4	128.3 ± 36.9	0.02
Dyslipidaemia, %	464 (70.8%)	415 (76.9%)	0.02
Medication intake, %			
Anti-diabetic	12 (1.8%)	22 (4.0%)	0.02
Anti-hypertensive	78 (11.8%)	94 (17.1%)	0.009
Energy intake, Kcal/day	2409.1 ± 919.4	2442.1 ± 958.1	0.58

Results are presented n (%) for qualitative variables and mean ± SD for quantitative variables

*p* Value from *X* ^2^ test and One way ANOVA for qualitative and quantitative outcomes respectively

BMI: Body Mass Index

With regard to selected health-related variables, in general, participants had better cardiometabolic profile compared to non-participants; in fact, prevalence of obesity (*P* < 0.0001), hypertension (P < 0.0001), diabetes (*P* = 0.007), as well as mean values of related biomarkers were significantly higher among non-participants.

Table 2 demonstrates a comparison of the overall ORISCAV-LUX2 sample (1558 subjects) according to the pathway of enrolment. In general, volunteers had a better health profile than other groups. The proportions of the sample are significantly different in terms of age, sex, and prevalence of main cardiometabolic risk factors. In the overall sample, prevalence estimates of diabetes, hypertension and obesity were 4.2, 30 and 19%, respectively).

**Table 2:** Comparison of the participant’s characteristics according to the strategy of enrolment, N = 1558 subjects

Variables	Baseline ORISCAV-LUX sample *N* = 660	EHES-LUX sample *N* = 455	Civil registry sample (CTIE) *N* = 269	Volunteers *N* = 174	Overall ORISCAV-LUX2 sample *N* = 1558	p value
Age, year	52.7 ± 12.0	49.1 ± 10.6	50.7 ± 12.7	48.7 ± 14.0	50.9 ± 12.1	< 0.0001
Sex, Men %	335 (50.8%)	191 (42.0%)	125 (46.5%)	81 (46.5%)	732 (47.0%)	0.04
District, %						
Diekirch	102 (15.5%)	75 (16.5%)	46 (17.1%)	24 (13.8%)	247 (15.9%)	0.39
Grevenmacher	92 (13.9%)	57 (12.5%)	37 (13.7%)	14 (8.1%)	200 (12.8%)	
Luxembourg	466 (70.6%)	323 (71.0%)	186 (69.1%)	136 (78.2%)	1111 (71.3%)	
Education level,%						< 0.0001
No diploma	115 (17.5%)	60 (13.3%)	34 (12.6%)	17 (9.8%)	226 (14.6%)	
Secondary level	302 (46.0%)	183 (40.4%)	110 (40.9%)	45 (25.9%)	640 (41.2%)	
University level	239 (36.4%)	210 (64.4%)	125 (46.5%)	112 (64.4%)	686 (44.2%)	
Marital status, %						0.07
Married	508 (85.1%)	326 (81.5%)	192 (80.3%)	129 (79.6%)	1155 (82.6%)	
Single	67 (11.2%)	61 (15.3%)	31 (13.0%)	29 (17.9%)	188 (13.5%)	
Divorced	8 (1.3%)	6 (1.5%)	3 (1.3%)	1 (0.6%)	18 (1.3%)	
Widowed	14 (2.4%)	7 (1.8%)	13 (5.4%)	3 (1.9%)	37 (2.7%)	
Country of birth,%						< 0.0001
Luxembourg	408 (61.8%)	272 (29.7%)	150 (16.4%)	86 (49.4%)	916 (58.8%)	
Portugal	64 (9.7%)	36 (7.9%)	13 (4.83%)	8 (4.6%)	121 (7.8%)	
Other European country	151 (22.9%)	115 (25.3%)	80 (29.74%)	70 (40.2%)	416 (26.7%)	
Non-European country	37 (5.6%)	32 (7.0%)	26 (9.7%)	10 (5.8%)	105 (6.7%)	
Physical activity, %						< 0.0001
Inactive	352 (53.9%)	127 (28.4%)	58 (21.6%)	35 (20.2%)	572 (37.1%)	
Moderately active	111 (17%)	104 (23.3%)	86 (32.1%)	60 (34.7%)	361 (23.4%)	
Active	190 (29.1%)	216 (48.3%)	124 (46.3%)	78 (45.1%)	608 (39.5%)	
Self-reported health perception, %						0.01
Very good	26 (4.0%)	20 (4.4%)	16 (6.0%)	10 (5.8%)	72 (4.6%)	
Good	156 (23.7%)	144 (31.9%)	85 (31.6%)	56 (32.4%)	441 (28.4%)	
Average	376 (57.2%)	241 (53.3%)	141 (52.4%)	87 (50.3%)	845 (54.5%)	
Bad	91 (13.9%)	37 (8.2%)	21 (7.8%)	18 (10.4%)	167 (10.8%)	
Very bad	8 (1.2%)	10 (2.2%)	6 (2.2%)	2 (1.2%)	26 (1.7%)	
Cardiometabolic Risk profile profile						
Smokers, %	86 (13.1%)	60 (13.5%)	36 (13.4%)	26 (15.3%)	208 (13.5%)	0.93
Diabetes, %	37 (6.3%)	9 (2.4%)	9 (3.7%)	2 (1.2%)	57 (4.2%)	0.005
Hypertension, %	225 (36.1%)	87 (22.7%)	80 (30.9%)	36 (21.6%)	428 (29.9%)	< 0.0001
Obesity, %	136 (22.4%)	72 (19.1%)	46 (18.1%)	15 (9.2%)	269 (19.2%)	0.002
BMI, kg/m^2^	26.9 ± 6.7	26.1 ± 4.9	26.1 ± 4.6	24.9 ± 4.3	26.3 ± 5.7	< 0.0001
Blood pressure, mmHg						
SBP	130.1 ± 16.7	125.5 ± 16.6	126.3 ± 16.7	123.1 ± 16.9	127.4 ± 16.9	< 0.0001
DBP	80.2 ± 9.1	77.4 ± 9.4	78.3 ± 9.5	75.5 ± 9.2	78.5 ± 9.4	< 0.0001
Serum glucose, mg/ml	96.0 ± 20.2	91.2 ± 13.4	92.4 ± 12.4	89.7 ± 23.0	93.3 ± 17.9	< 0.0001
Serum cholesterol, mg/dl	204.6 ± 37.7	206.6 ± 39.1	203.5 ± 37.8	203.7 ± 35.9	204.8 ± 37.9	0.87

Results are presented n (%) for qualitative variables and mean ± SD for quantitative variables

p Value from *X* ^2^ test and t test for qualitative and quantitative outcomes respectively

*CTIE Centre des Technologies de l’Information de l’Etat*, *BMI* Body Mass Index

To assess the representativeness, the overall ORISCAV-LUX2 sample (1558 participants) was compared to the Luxembourg population (342,235 individuals, National Institute of Statistics, STATEC 2011) according to the stratification criteria: sex, age category and district of residence. Table 3 shows that ORISCAV-LUX 2 sample was representative of the population for district, but not for sex and age groups. This age difference was significant for both men and women (both P < 0.0001). Compared to the Luxembourg population, the younger (25–34 years) and older (65–79 years) age groups were underrepresented, whereas middle-aged adults (45–64) were over-represented in the overall sample.

**Table 3:** Comparison of ORISCAV-LUX2 participants to the Luxembourg population by sex, age category and district of residence

Stratification criteria	Luxembourg population	Participants^a^	p value
	(*N* = 342,235)	(*N* = 1556)	
	n (%)	n (%)	
Sex			0.02
Men	171,158 (50.0%)	730 (46.9%)	
Women	171,077 (50.0%)	826 (53.1%)	
Age category (years)			
Women			< 0.0001
25–34 years	36,895 (21.6%)	96 (11.6%)	
35–44 years	40,575 (23.7%)	185 (22.4%)	
45–54 years	37,906 (22.2%)	235 (28.4%)	
55–64 years	27,725 (16.2%)	202 (24.5%)	
65–79 years	27,976 (16.3%)	108 (13.1%)	
Men			< 0.0001
25–34 years	36,625 (21.4%)	89 (12.2%)	
35–44 years	41,519 (24.3%)	175 (24.0%)	
45–54 years	40,216 (23.5%)	196 (26.8%)	
55–64 years	28,760 (16.8%)	176 (24.1%)	
65–79 years	24,038 (14.0%)	94 (12.9%)	
District			0.17
Luxembourg	251,601 (73.5%)	1111 (71.4%)	
Diekirch	50,207 (14.7%)	246 (15.8%)	
Grevenmacher	40,427 (11.8%)	199 (12.8%)	

^a^There were 2 participants having 80 or more years old, they were excluded

Table 4 shows the completeness of individual survey elements. Data from the self-administered questionnaires were fully available, including 65% completed online, and 35% completed on paper. The percentage of completeness for health questionnaires, e-FFQ questionnaires and clinical and anthropometric measurements varied between 90 to 92%. Physical function measurements (Actigraph® and Actiheart®) were lowest (76 and 65%, respectively). The samples of biological material; blood, urine and hair were all available for 89, 85 and 55% of the participants, respectively.

**Table 4:** Completeness of individual survey elements

Survey elements	Completeness n (%)
Self-administered questionnaire	1557 (99.9%)^a^
Online	1008 (64.7%)
By paper	549 (35.3%)
Interview with research nurse	
Health questionnaire (global)	1438 (92.3%)
e-FFQ	1432 (91.9%)
ADL/IADL	1437 (92.2%)
Anthropometry	1434 (92.0%)
Blood pressure	1435 (92.1%)
CANTAB®	1419 (91.1%)
MMSE-2	1436 (92.2%)
Tanita®	1402 (90.0%)
ECG	1428 (91.7%)
Complior®	1404 (90.1%)
Physical tests (finger tapping test, balance test, grip strength test, chair rising test)	1431 (91.8%)
Step test / Actiheart®	1006 (64.6%)
Accelerometry /Actigraph®	1190 (76.4%)
Blood samples	1382 (89.0%)
Urine samples	1329 (85.3%)
Hair samples	857 (55.0%)

^a^One participant attended the interview but refused to fill-in the self-reported questionnaire

## Discussion

Principal investigators of population surveys face big challenges to manage the data collection as planned and need to create opportunities to adapt the design during the course of data collection in order to ensure quality and external validity of constituted datasets and hence the resulting statistics.

The present manuscript highlights the implementation of adaptive sampling schemes based on our experience in setting up the second wave of the ORISCAV-LUX survey. Indeed, enrolment of the same participants nine years later seemed a highly intricate task. Extensive efforts were required to search and locate former participants in baseline study. A total of 1209 addresses were identified and invited, including 660 subjects (55%) were successfully enrolled. However, it was crucial to recruit additional subjects and implement further alternative strategies to increase the sample size and enhance national representativeness, including random sampling and call for volunteers.

Consistent to most literature supporting the notion of “healthy participant bias” [10–13], our findings reported that baseline participants in the ORISCAV-LUX2 study were generally healthier and at less risk than those who refused to take part. However, examples of non-significant differences [14, 15] or opposite findings have also been reported [16, 17]. Likewise, the respondents to our invitations were of higher education level than the non-respondents [12, 18, 19]. Such difference and low response rate may imply greater potential for bias survey estimates [20, 21]. In addition, this study confirmed differences in the socio-economic characteristics and cardiometabolic health profile of subjects enrolled via the different pathways, although the major proportion of the overall ORISCAV-LIX2 sample were randomly selected (baseline, EHES-LUX and CITE).

Using an additional list of subjects’ addresses was also used in a similar German population-based study [22], with relevant conclusions. Convenience sampling is affordable, and the subjects are readily available. As confirmed by our study, people who volunteer tend to be more health conscious than others [23]. Therefore, samples based only on volunteers are not likely to be representative of the general population, threatening hence the generalisability of the study results. This small volunteers’ segment could be excluded from future analyses according to specific research objectives and if deemed necessary after secondary analyses.

With these corrective measures, we raised the number of participants up to 1558, including 1438 subjects (92.3%) with full participation (filled in questionnaire and attended appointment with the research nurse). Indeed, this is an utmost advantage for the credibility of future analyses on the ORISCAV-LUX2 dataset, targeting prevalence estimates, for example, cognitive performance, arterial stiffness and physical disability.

In observational epidemiology, in particular for studies with a follow-up design, it is important to distinguish scientific inference from population inference [24]. Goldstein et al. [24] suggested to make a clear distinction between descriptive statistics that require representative samples and analytical statistics that attempt to address scientific hypotheses. They argued that selecting a sample that does not represent a real population but has a high degree of heterogeneity in terms of outcome, may provide much more power to investigate the hypotheses of interest. Therefore, they concluded that heterogeneity is desirable to enhance the effectiveness of analysis, and this often implies using sample that is not necessarily representative of the real population [24]. In addition, most of the etiological research on chronic disease (including cardiovascular diseases) issued from highly selected populations with limited representativeness, for example the Framingham study [25] and the Whitehall studies in the UK [26].

Compared to the Luxembourg population, the ORISCAV-LUX2 sample was representative for district of residence, but not for sex and age, with the younger (25–34 years) and older (65–79 years) age groups being underrepresented, whereas middle-aged adults (45–64 years) were over-represented. In the ORISCAV-LUX2 study, high coverage and sample representativeness is the primary purpose for adopting this hybrid sampling frame as an alternative solution to only use the baseline sample. Interestingly, this initial analyses of the total sample demonstrated that the prevalence of diabetes, hypertension and obesity are comparable to that reported in 2007–2008 (4.2, 30 and 19%, respectively) [2]. Assuming a steady pattern, this would indicate that integrating diverse sampling strategies in the second wave would not have biased our approach to assess the trend of these disease conditions nine years later. Nevertheless, a number of measures will be considered in future analyses in order to ensure population inference [24]. These include post-survey adjustment of data using weighting techniques to correct for non-response bias [27], as well as using statistical models based on the characteristics of the initial respondents to ‘adjust’ subsequent analyses [24, 28].

It is worth noting that strict control measures were applied to ensure quality throughout the conduct of the study. Intensive efforts were provided to optimally prepare the fieldwork including nurses training to standard operating procedures. Several features in the survey process would affect response rate and the type of participation (full vs. partial), such as the way and number of contacts, type of information given to the participants, language of the communication documents, length of interview and feedback received on examination results. While the mean time needed to perform the first wave appointments was less than 2 h, the time for the second wave appointment varied from minimum 01:55 to a maximum of 06:15 (with a mean duration of 03 h:17 min). Based on the 1438 participants who were interviewed, the completeness of individual survey elements can be described as optimal.

## Conclusion

This study represents a careful first-stage analysis of the ORISCAV-LUX2 sample, based on available information on participants and non-participants. It stresses that special adaptive procedures in sampling design are needed to gain an optimal sample size. These procedures may provide the only practical way to obtain a sample large enough for both scientific research objectives and population inference. A central issue for success of observational studies is to achieve an appropriate balance between adapting the initial sampling procedure during data collection and a later adjustment with sample weighting. The available ORISCAV-LUX datasets provide a relevant basis for policy-makers regarding public health monitoring and evidence-based prevention, as well as constitute a valuable tool for epidemiological research on cardiometabolic risk.

## Additional files

Additional file 1: Questionnaires ORISCAV-LUX 2. (Nurse Questionnaire) English version. (PDF 872 kb)

Additional file 2: Questionnaires ORISCAV-LUX 2. (Home-based Questionnaire) English version. (PDF 898 kb)

## Abbreviations

ADL: Activities of Daily Living
ANOVA: Analysis of variance
BMI: Body Mass Index
CANTAB: Cambridge Neuropsychological Test Automated Battery
CES-D: Centre for Epidemiologic Studies Depression Scale
CITE: *Centre des Technologies de l’Information de l’Etat*
e-FFQ: Electronic Food Frequency Questionnaire
EHES-LUX: European Health Examination Survey in Luxembourg
IADL: Instrumental Activities of Daily Living
IGSS: General Inspectorate of Social Security
LIH: Luxembourg institute of Health
MMSE-2: Mini-Mental State Examination test-version 2
ORISCAV-LUX: Observation of Cardiovascular risk factors study in Luxembourg (Baseline)
ORISCAV-LUX2: Second wave of the Observation of Cardiovascular risk factors study in Luxembourg
PASW: Predictive Analytics Software
SD: Standard deviation
SF-36: Quality of life 36-Item Short Form Health Survey
STATEC: *Institut national de la statistique et des études économiques du Grand-Duché de Luxembourg*
