# Supplementary material for: Challenges and benefits of integrating diverse sampling strategies in the observation of cardiovascular risk factors (ORISCAV-LUX 2) study
Source: BMC Med Res Methodol. 2019 Feb 4;19:27. doi: 10.1186/s12874-019-0669-0 (PMC6360765; doi:10.1186/s12874-019-0669-0)
Supplement: Supplementary file 1 — Questionnaires ORISCAV-LUX 2. (Nurse Questionnaire) English version. (PDF 872 kb) [file 12874_2019_669_MOESM1_ESM.pdf]

STUDY NURSE QUESTIONNAIRE

Dear Madam, Dear Sir,

Thank you that you have already filled in the first questionnaire.

The study nurse will explain you the progress of this visit.

1. WELCOME

Date of the appointment: ☐☐ / ☐☐ / ☐☐☐☐
Day Month Year

Starting time: ☐☐ : ☐☐
Hours : Minutes

Initials of the study nurse: ☐☐☐

WELCOME CHECK LIST

Yes	No	
☐	☐	The participants identity (name, birthday, gender) and address checked.
☐	☐	Information letter and informed consent read and explained to the participant with advantages and risks.
☐	☐	Consent form signed in 2 copies.
☐	☐	Home-based questionnaire validated with the participant.

If no, for what reason:

☐	Forgotten by the participant.
☐	Home-based questionnaire is not completed. It is given back with postage-paid envelope.
☐	Refusal of filling in.

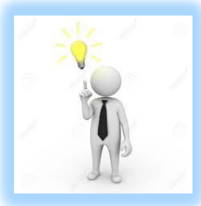

IF POSSIBLE, GIVE THE PARTICIPANT NOTHING TO DRINK BETWEEN THE WELCOMING AND THE IMPEDANCEMETRY.

2. COGNITION

2.1. CANTAB (Cambridge Neuropsychological Test Automated Battery)

Five tests of the battery CANTAB are carried out:
1. Motor Screening,
2. Paired Associates Learning,
3. Reaction Time,
4. Spatial Working Memory,
5. Rapid Visual Information Processing.

2.1.1. ID of device: ☐

2.1.2. The test has been carried out alone by the participant on a touchscreen:

☐ Yes
☐ No

2.1.3. The participant has forgotten his glasses, the study nurse lend him reading glasses:

☐ Yes
☐ No
☐ Not applicable

2.1.4. Reason why the test was not carried out

☐ Refusal
☐ Anxiety/Fear
☐ Parkinson's disease, Tremor
☐ Glasses forgotten, no glasses suitable
☐ Test not understood
☐ Other, specify : ....

2.1.5. Observations of the study nurse

....
....
....
....

2.2. MMSE-2 (Mini-Mental State Examination)

The study nurse conducts the examination with the participant in a quiet setting. She/he gives him sufficient time for responses. Do not hesitate to reinforce positively good answers and to minimise errors.

"I will ask you some questions in order to assess your memory skills. Some are very simple, others a little less. You have to respond as best as you can."

2.2.1. The test has been realised

☐ Yes
☐ No

2.2.2. Reason why the test was not carried out

☐ Refusal
☐ Other, specify: ....

The study nurse stapples the MMSE-2 test results on the next page.

2.2.3. Assessment of level of consciousness

☐ Alert/responsive
☐ Drowsy
☐ Stuporous
☐ Comatose/unresponsive

Now I would like to ask you some questions about your memory.

REGISTRATION

2.2.4. Listen carefully. I am going to say three words. You say them back after I stop. Ready? Here they are… MILK [*pause*], SENSIBLE [*pause*], BEFORE [*pause*]. Now repeat those words back to me. [*Repeat up to 3 times, but score only the first trial*.]

3 words	Response of the participant	Correct	Wrong
MILK		☐	☐
SENSIBLE		☐	☐
BEFORE		☐	☐

Now keep those words in mind. I am going to ask you to say them again in a few minutes.

ORIENTATION TO TIME

2.2.5. What day is today? What is …

	Response of the participant	Correct	Wrong
The year?		☐	☐
The season?		☐	☐
The month of the year?		☐	☐
The day of the week?		☐	☐
The date?		☐	☐

SPECIMEN

ORIENTATION TO PLACE

2.2.6. What day is today? What is …

Alternative place words that are appropriate for the setting and increasingly precise may be substituted and noted.

	Response of the participant	Correct	Wrong
State (or province)?		☐	☐
County (or city/town)?		☐	☐
City/town (or part of city/ neighborhood)?		☐	☐
Building (name or type)?		☐	☐
Floor of the building (room number or address)?		☐	☐

RECALL

2.2.7. What were those three words I asked you to remember?

The nurse does not offer any hints.

3 words	Response of the participant	Correct	Wrong
MILK		☐	☐
SENSIBLE		☐	☐
BEFORE		☐	☐

SPECIMEN

3. AUTONOMY

3.1. ADL - Echelle de Katz: Independence in Activities of Daily Living

3.1.1.

	**NO** supervision, direction or personal assistance	**WITH** supervision, direction, personal assistance or total care
Bathing	☐ Bathes self completely or needs help in bathing only a single part of the body such as the back, genital area or disabled extremity.	☐ Needs help with bathing more than one part of the body, getting in or out of the tub or shower. Requires total bathing.
Dressing	☐ Get clothes from closets and drawers and puts on clothes and outer garments complete with fasteners. May have help trying shoes.	☐ Needs help with dressing self or needs to be completely dressed.
Toileting	☐ Goes to toilet, gets on and off, arranges clothes, cleans genital area without help.	☐ Needs help transferring to the toilet, cleaning self or uses bedpan or commode.

	**NO** supervision, direction or personal assistance	**WITH** supervision, direction, personal assistance or total care
Transferring	☐ Moves in and out of bed or chair unassisted. Mechanical transferring aides are acceptable.	☐ Needs help in moving from bed to chair or requires a complete transfer.
Continence	☐ Exercises complete self control over urination and defecation.	☐ Is partially or totally incontinent of bowel or bladder.
Feeding	☐ Gets food from plate into mouth without help. Preparation of food may be done by another person.	☐ Needs partial or total help with feeding or requires parenteral feeding.

3.2. IADL - Echelle de LAWTON: Instrumental Activities of Daily Living Scale

3.2.1. Ability to Use Telephone

☐	Operates telephone on own initiative; looks up and dials numbers.
☐	Dials a few well-known numbers.
☐	Answers telephone, but does not dial.
☐	Does not use telephone at all.

3.2.2. Shopping

☐	Takes care of all shopping needs independently.
☐	Shops independently for small purchases.
☐	Needs to be accompanied on any shopping trip.
☐	Completely unable to shop.

3.2.3. Food Preparation

☐	Plans, prepares and serves adequate meals independently.
☐	Prepares adequate meals if supplied with ingredients.
☐	Heats and serves prepared meals or prepares meals but does not maintain adequate diet.
☐	Needs to have meals prepared and served.

3.2.4. Housekeeping

☐	Maintains house alone with occasion assistance (heavy work).
☐	Performs light daily tasks such as dishwashing, bed making.
☐	Performs light daily tasks, but cannot maintain acceptable level of cleanliness.
☐	Needs help with all home maintenance tasks.
☐	Does not participate in any housekeeping tasks.

3.2.5. Laundry

☐	Does personal laundry completely.
☐	Launders small items, rinses socks, stockings, etc.
☐	All laundry must be done by others.

3.2.6. Mode of Transportation

☐	Travels independently on public transportation or drives own car.
☐	Arranges own travel via taxi, but does not otherwise use public transportation.
☐	Travels on public transportation when assisted or accompanied by another.
☐	Travel limited to taxi or automobile with assistance of another.
☐	Does not travel at all.

3.2.7. Responsibility for Own Medications

☐	Is responsible for taking medication in correct dosages at correct time.
☐	Takes responsibility if medication is prepared in advance in separate dosages.
☐	Is not capable of dispensing own medication.

3.2.8. Ability to Handle Finances

☐	Manages financial matters independently (budgets, writes checks, pays rent and bills, goes to bank); collects and keeps track of income.
☐	Manages day-to-day purchases, but needs help with banking, major purchases, etc.
☐	Incapable of handling money.

4. HEALTH

4.1. PERSONAL MEDICAL HISTORY

4.1.1. Which diseases or health discomfort do you currently have?

	Yes	No	Disease diagnosed by a medical doctor?		Disease in treatment?	
			Y	N	Y	N
Heart failure	☐	☐	☐	☐	☐	☐
Cardiac arrhythmia	☐	☐	☐	☐	☐	☐
Angina pectoris	☐	☐	☐	☐	☐	☐
Myocardial infarction (heart attack)	☐	☐	☐	☐	☐	☐
Valvular heart defect	☐	☐	☐	☐	☐	☐
Stroke	☐	☐	☐	☐	☐	☐
High blood pressure (hypertension)	☐	☐	☐	☐	☐	☐
Arteritis of the lower extremity	☐	☐	☐	☐	☐	☐
Varicose veins, venous insufficiency	☐	☐	☐	☐	☐	☐
Ulcerated legs	☐	☐	☐	☐	☐	☐
Chest pain or shortness of breath on exertion	☐	☐	☐	☐	☐	☐
Diabetes:<br>Diabetes Typ 1 ☐<br>Diabetes Typ 2 ☐	☐	☐	☐	☐	☐	☐
Dyslipidemia	☐	☐	☐	☐	☐	☐
Cancer<br>Which:	☐	☐	☐	☐	☐	☐

	Yes	No	Disease diagnosed by a medical doctor?		Disease in treatment?	
			Y	N	Y	N
Kidney problems	☐	☐	☐	☐	☐	☐
Psoriasis	☐	☐	☐	☐	☐	☐
Psoriatic arthritis	☐	☐	☐	☐	☐	☐
Gout	☐	☐	☐	☐	☐	☐
Rheumatoid arthritis	☐	☐	☐	☐	☐	☐
Ankylosing spondylitis	☐	☐	☐	☐	☐	☐
Crohn's disease	☐	☐	☐	☐	☐	☐
Ulcerative colitis	☐	☐	☐	☐	☐	☐

4.1.2. Have you experienced **in the course of the last year** one or several of the following events?

Yes	No	
☐	☐	The death of a person close to you
☐	☐	A personal, serious disease
☐	☐	A serious disease of a person close to you
☐	☐	A personal admission at hospital
☐	☐	A divorce
☐	☐	A traffic accident
☐	☐	A crime

➔If the participant is a man, SKIP TO chapter MEDICATION AND SUPPLEMENTS on page 17

4.2. WOMEN'S HEALTH

4.2.1. Do you have a cycle?

☐ Yes
☐ No

If not, have you had one of the following surgeries at any time in your life:

	Yes	No
Hysterectomy	☐	☐
Bilateral ovariectomy	☐	☐

4.2.2. Do you have children?

☐ Yes
☐ No

If yes, how many children do you have?

☐☐ Child(ren)

4.2.3. Do you currently breastfeed or have you breastfed in the past?

☐ Yes
☐ No

If yes, complete the following table:

	Duration (months)	Not applicable
Current breastfeeding	☐☐	☐
Child 1 breastfeeding	☐☐	☐
Child 2 breastfeeding	☐☐	☐
Child 3 breastfeeding	☐☐	☐
Child 4 breastfeeding	☐☐	☐
Child 5 breastfeeding	☐☐	☐
Child 6 breastfeeding	☐☐	☐
Child 7 breastfeeding	☐☐	☐

4.2.4. Are you currently going through menopause?

☐ Yes
☐ No
☐ I do not know

➔GO to chapter MEDICATION AND SUPPLEMENTS on page 17

If yes, what is the date of your last menstruations?

☐☐ / ☐☐ / ☐☐☐☐
Day Month Year

4.2.5. Do you currently follow a treatment for the menopause?

☐ Yes
☐ No
☐ I do not know

➔ GO to chapter MEDICATION AND SUPPLEMENTS on page 17

4.2.6. Which type of treatment is it?

	Yes	No
Hormones	☐	☐
Vaginal local treatment	☐	☐
Non hormonal treatment against hot flashes	☐	☐
Homeopathy	☐	☐
Plants	☐	☐
➔**If yes**, are they phytoestrogens (e.g. soya)?	☐	☐
Acupuncture	☐	☐
Other treatment, specify :	☐	☐
I do not know	☐	☐

4.3. MEDICATION AND SUPPLEMENTS

4.3.1. Do you currently take medications and/or supplements?

☐ Yes
☐ No ➔ **SKIP TO chapter VITAMINS SUPPLY on page 19**

4.3.2. Did you bring your medicine packages or a prescription and/or a list with the **supplements** with you?

☐ Yes
☐ No

4.3.3. Would you allow me to have a look and write down which medicines you are taking?

☐ Yes
☐ Refusal
☐ Forgotten or missing data

Ask the person if she regularly takes sleeping pills.

Name	Galenic form	Dosage	Number of dosage units	Frequency	Duration
i.e. ASPIRIN	TABLET	500 MG	2	1-0-1-0	5 DAYS/ 3 MONTHS/ 7 YEARS

Name	Galenic form	Dosage	Number of dosage units	Frequency	Duration

4.4. VITAMINS SUPPLY

4.4.1. Do you regularly take vitamins, minerals or other supplements?

- ☐ Yes
- ☐ No → **SKIP TO chapter NUTRITION on page 20**

If yes, do you take:

Yes	No	
☐	☐	Multivitamins (WITH minerals)
☐	☐	Multivitamins (WITHOUT minerals)
☐	☐	Fish oil (including cod liver)
☐	☐	Garlic
☐	☐	Iron
☐	☐	Zinc
☐	☐	Calcium
☐	☐	Vit A
☐	☐	Vit B (including B6 and B12)
☐	☐	Vit C
☐	☐	Vit D
☐	☐	Vit E

4.4.2. Do your vitamin tablets contain folic acid?

- ☐ Yes
- ☐ No
- ☐ I do not know

5. NUTRITION

5.1. FFQ ONLINE (Food Frequency Questionnaire)

The past 3 months,
What type of food and drink have you regularly consumed and in what quantities?

Try to think about your eating habits during the week and on weekends, at home and in the restaurant.

A manual with photos will guide you through the filling in.

6. CONDITIONS OF REALISING EXAMINATIONS

6.1.1. Room temperature

☐☐,☐ °C

6.1.2. Has the participant done any of the following activities **1 hour before the examination**?

Smoked	☐ Yes	☐ No
Drank something else than water	☐ Yes	☐ No

6.1.3. Before starting the anthropometry, the study nurse checks the following conditions together with the participant:

	Yes	No	Time	Not applicable
Alcohol consumption in the last 12 hours	☐	☐	___ h ___	☐
Vigorous exercise less than 12 hours before the measurement	☐	☐	___ h ___	☐
Excessive intake of food and drink on the day of measurement	☐	☐	___ h ___	☐
Ate and drank in the last 3 hours before measurement	☐	☐	___ h ___	☐
Urinated right before measurement (advice)	☐	☐	___ h ___	☐

7. ANTHROPOMETRY

7.1. BODY MEASUREMENTS

HEIGHT

7.1.1. ID number of device: ☐

7.1.2. Measurement of the height

☐☐☐ , ☐ cm

☐ Not measured

7.1.3. Reason why the size has not been measured

☐ Refusal
☐ Other, specify: ....

WEIGHT

7.1.4. In the past 12 months, have you lost **unintentionally** at least 5 kg (**not because** of a diet or physical activity)?

☐ Yes
☐ No

WAISTSIZE

7.1.5. ID number of device: ☐

7.1.6. Waist size:

☐☐☐ , ☐ cm

☐ Not measured

7.1.7. The measurement has been made

☐ **Directly on the skin**
☐ In underwear
☐ On light clothing, specify: ....
☐ Other, specify: ....

7.1.8. Reason why the waist size has not been measured

☐ Refusal
☐ Other, specify: ....

HIP SIZE

7.1.9. Hip size:

☐☐☐ , ☐ cm

☐ Not measured

7.1.10. The measurement has been made

☐ **In underwear**

☐ On light clothing, specify: ....

☐ Other, specify: ....

7.1.11. Reason why the hip size has not been measured

☐ Refusal

☐ Other, specify: ....

THIGH SIZE

7.1.12. **PROXIMAL** left thigh size

☐☐☐ , ☐ cm

☐ Not measured

7.1.13. The measurement has been made

☐ **Directly on the skin**
☐ On light clothing, specify: ....
☐ Other, specify: ....

7.1.14. Which thigh has been measured?

☐ Left
☐ Right

If the right thigh has been measured, specify the reason:
....

7.1.15. Reason why the thigh size has not been measured

☐ Refusal
☐ Other, specify: ....

7.2. IMPEDANCEMETRY

Specific vocabulary for this measurement:
FM - Masse grasse
FFM - Masse maigre
PMM - Masse musculaire

7.2.1. ID of device: ☐

7.2.2. Before starting, the study nurse checks these exclusion criteria:

	Yes	No	Not applicable
Period by the woman (exclusion)	☐	☐	☐
Wearing a pacemaker	☐	☐	////////////////

➔If yes, MEASURE the weight of the SECA scale and CONTINUE.
➔If no/not applicable, SKIP TO question 7.2.7. on the following page.

7.2.3. ID of device: ☐

7.2.4. Weight

☐☐☐ , ☐☐ kg

7.2.5. The measurement has been made

- ☐ **In underwear**
- ☐ With light clothing, specify: ....
- ☐ Other, specify: ....

7.2.6. Reason why the weight has not been measured

- ☐ Wheelchair or immobile
- ☐ Varies standing
- ☐ Refusal
- ☐ Other,specify: ....

➔SKIP To chapter CARDIOLOGY on page 30.

7.2.7. Time of measurement

☐☐ : ☐☐

Hours : Minutes

7.2.8. The analysis has been made

☐ **In underwear, without shoes and without jewelry**

☐ With light clothing, specify: ....

☐ Other, specify: ....

7.2.9. Reason why the analysis was not carried out

☐ Refusal

☐ Other, specify: ....

The study nurse staples the results of the impedancemetry on page 2

8. CARDIOLOGY

8.1. BLOOD PRESSURE

8.1.1. **In the past 12 months,** how often was your blood pressure measured?

☐☐ / day

☐☐ / week

☐☐ / month

☐☐ / year

Before the first measurement, the participant must remain seated and quiet for at least 5 minutes.

8.1.2. Type of device

☐ OMRON MX3 PLUS

☐ OMRON M6 COMFORT

ID of device: ☐

8.1.3. Selection of cuff

Arm measurement: ☐☐ , ☐ cm

Cuff used:

☐ Small-Medium for OMRON MX3 PLUS (Circumference of the arm 22-32 cm)

☐ Large for OMRON MX3 PLUS (Circumference of the arm 32-42 cm)

☐ Small-Medium-Large for OMRON M6 COMFORT (Circumference of the arm 22-42 cm)

SPECIMEN

8.1.4. Measurement of blood pressure on the **RIGHT arm**

Arm used: ☐ Right ☐ Left

1 minute pause between each measurement.

	1st	2nd	3rd
Systolic pressure (mmHg):	☐☐☐	☐☐☐	☐☐☐
Diastolic pressure (mmHg):	☐☐☐	☐☐☐	☐☐☐
Pulse (/60 sec):	☐☐☐	☐☐☐	☐☐☐
No measurement:	☐	☐	☐
Error code of device:	....	....	....

8.1.5. If you used the **LEFT arm**, give the reason

- ☐ Ganglionic problem affecting the right arm (e.g. after breast cancer)
- ☐ Right arm paralysed or spastic
- ☐ Amputation of the right arm
- ☐ Right arm in plaster
- ☐ Other, specify: ....

8.1.6. Position of the participant during blood pressure measurement

- ☐ Sitting
- ☐ Lying down

8.1.7. If the participant is lying, give the reason:

....

....

8.2. CARDIOVASCULAR HISTORY

8.2.1. Are you followed by a cardiologist?

- ☐ Yes
- ☐ No ➔ **SKIP TO the question 8.2.5.**

8.2.2. If yes, do you know why you are followed by a cardiologist?

....

....

8.2.3. When was the last time you consult with your cardiologist?

☐☐/☐☐/☐☐☐☐

Day Month Year

8.2.4. What is the name of **your cardiologist**, so the cardiologist of the study can contact him if your ECG would be significantly abnormal?

Dr (NAME):

Forename:

Practice:

☐ Refusal of participant

8.2.5. What is the name of **your family doctor**, so the cardiologist of the study can contact him if your ECG would be significantly abnormal?

Dr (NAME):

Forename:

Practice:

☐ Refusal of participant

8.2.6. Did a doctor diagnose you one of the following diseases?

(Look at Page 13 and 14)	Yes	No	I do not know	Date
Coronary heart disease, Myocardial infarction	☐	☐	☐	___/ ___/____
Valvular heart defect	☐	☐	☐	___/ ___/____
High blood pressure	☐	☐	☐	___/ ___/____
Chronic lung disease, Asthma	☐	☐	☐	___/ ___/____
Diabetes	☐	☐	☐	___/ ___/____
Renal insufficiency	☐	☐	☐	___/ ___/____
Anemia	☐	☐	☐	___/ ___/____
Acute rheumatic fever in childhood	☐	☐	☐	___/ ___/____
Other heart disease, describe please:	☐	☐	☐	___/ ___/____

8.2.7. Did you have one of the following family histories?

	Yes	No	I do not know	Date
Myocardial infarction <55 years at father or brother	☐	☐	☐	___/ ___/____
Myocardial infarction <65 years at mother or sister	☐	☐	☐	___/ ___/____
Stroke <45 years at parents or at brother or sister	☐	☐	☐	___/ ___/____
High blood presure	☐	☐	☐	___/ ___/____
High cholesterol	☐	☐	☐	___/ ___/____
Diabetes	☐	☐	☐	___/ ___/____

8.2.8. Let us return on to your habits and your lifestyle:

How many cigarettes do you smoke?	☐☐<br>☐☐	/ day<br>/ week
How many glasses of alcohol do you drink?	☐☐<br>☐☐	/ day<br>/ week
How many times a week do you practice a physical activity?	☐☐	/ week

8.2.9. Do you currently have the following symptoms?

	Yes	No
Chest pain	☐	☐
Dyspnoea/shortness of breath on exertion	☐	☐
Arrhytmia	☐	☐
Syncope, faintness	☐	☐

8.3. ELECTROCARDIOGRAM

8.3.1. ID number of device: ☐

8.3.2. ECG has been made

☐ Yes
☐ No

If no, give the reason:

☐ Refusal
☐ Other, specify : ....

8.3.3. If the participant wears a pacemaker, did you use a magnet in the implementation of the ECG?

☐ Yes
☐ No
☐ Not applicable

If no, give the reason:

☐ Refusal
☐ Other, specify: ....

8.4. PULSE WAVE VELOCITY

It is an examination which allows to measure the arterial rigidity by the analysis of the speed of wave pulse and the central pressure. During the whole investigation, the couch remains in a horizontal position, without pillow and without folded up back part. The resting period before carrying out the examination is 10 minutes.

8.4.1. ID number of device: ☐

8.4.2. The carotid-femoral PWV measurement has been carried out?

☐ Yes
☐ No

If no, give the reason:
☐ Refusal
☐ Other, specify: ....

8.4.3. What is the distance between the carotid and the femoral arteries?

☐☐☐ mm

8.4.4. Blood pressure is measured 3 times on the right arm during the examination:

	1st	2nd	3rd
Systolic pressure (mmHg):	☐☐☐	☐☐☐	☐☐☐
Diastolic presure (mmHg):	☐☐☐	☐☐☐	☐☐☐
Pulse (/60 sec):	☐☐☐	☐☐☐	☐☐☐
No measurement:	☐	☐	☐
Error code of device:	....	....	....

9. PHYSICAL FITNESS

10.1. FINGER TAPPING TEST

*The participant is asked to touch the sensor as fast as possible, for a period of 10 seconds. The hand is placed flat and the fingertips touch the board. It has to start with the **dominant hand** and then the non-dominant hand.*

10.1.1. ID of device: ☐

10.1.2. The participant is:

☐ Right hander
☐ Left hander

10.1.3. The number of keys counted by hand:

Dominant hand

1.	☐☐☐	keys/10’’
2.	☐☐☐	keys/10’’
3.	☐☐☐	keys/10’’

NON dominant hand

1.	☐☐☐	keys/10’’
2.	☐☐☐	keys/10’’
3.	☐☐☐	keys/10’’

10.1.4. Reason why the measurement of the **RIGHT** hand has not been performed:

☐ Amputation: arm, hand
☐ Paralysis of the arm, the hand
☐ Plaster
☐ Bandages
☐ Operation within the 3 past months
☐ Other missing fingers or fingers broken
☐ Refusal
☐ Other, specify: ....

10.1.5. Reason why the measurement of the **LEFT** hand has not been performed:

- ☐ Amputation: arm, hand
- ☐ Paralysis of the arm, the hand
- ☐ Plaster
- ☐ Bandages
- ☐ Operation within the 3 past months
- ☐ Other missing fingers or fingers broken
- ☐ Refusal
- ☐ Other, specify: ....

10.2. GRIP STRENGTH TEST

The study nurse demonstrates the participant the test and then gives him an opportunity to try the dynamometer itself. In this way, the study nurse tests the understanding of the participant and can adjust the handle size.

10.2.1. ID number of device ☐

10.2.2. Have you had, **in the past 7 days**, pain or a feeling of stiffness in the **RIGHT** hand?

☐ Yes
☐ No
☐ Not applicable

10.2.3. Have you had, **in the past 7 days**, pain or a feeling of stiffness in the **LEFT** hand?

☐ Yes
☐ No
☐ Not applicable

10.2.4. Which is your dominant hand?

☐ Right
☐ Left

10.2.5. Three measurements per hand are provided:

1 minute pause between each measurement.

NON dominant hand

1.	☐☐☐	kg
2.	☐☐☐	kg
3.	☐☐☐	kg

Dominant hand

1.	☐☐☐	kg
2.	☐☐☐	kg
3.	☐☐☐	kg

10.2.6. In which position, the measurements have been carried out?

☐	**Standing**
☐	Sitting<br>**If SITTING**, give the reason:<br>...

10.2.7. Reason why the measurement of the **RIGHT** hand has not been performed:

☐ Amputation: arm, hand, thumb
☐ Paralysis of the arm, the hand
☐ Plaster
☐ Bandages
☐ Operation within the 3 past months
☐ Other missing fingers or fingers broken
☐ Refusal
☐ Other, specify: ....

10.2.8. Reason why the measurement of the **LEFT** hand has not been performed:

☐ Amputation: arm, hand, thumb
☐ Paralysis of the arm, the hand
☐ Plaster
☐ Bandages
☐ Operation within the 3 past months
☐ Other missing fingers or fingers broken
☐ Refusal
☐ Other, specify: ....

10.3. BALANCE TEST

The study nurse performs a demonstration. The test is carried out without technical assistance (cane, assistant, etc.). The participant wears preferably low-heeled shoes.

10.3.1. Do you, in your daily life, have any problems with seeing?

Even when wearing your glasses or contact lenses, if you are concerned.

☐ Yes
☐ No

10.3.2. Do you, in your daily life, have any problems with hearing?

Even when using your hearing aid, if you are concerned.

☐ Yes
☐ No

10.3.3. Does the participant have a physical deformity preventing from joining both feet?

Yes	No	
☐	☐	Genu valgum (X position)
☐	☐	Hallux valgus
☐	☐	Strong thighs

10.3.4. **For the "feet together" position**, does the participant succeed in keeping all alone the position?

☐ Yes
☐ No ➔**SKIP TO question 10.3.22. on page 49**

10.3.5. **"Feet together"** for **10 seconds**:

☐☐ , ☐☐ seconds

☐ Successful test
☐ Not successful test ➔ **SKIP TO question 10.3.22. on page 49**

10.3.6. Was the participant likely to fall without the help of the nurse?

☐ Yes
☐ No

10.3.7. . **For the "semi-tandem" position**, does the participant manage alone to keep the position?

☐ Yes
☐ No ➔**SKIP TO question 10.3.22. on page 49**

10.3.8. **„Semi-tandem"** for 10 seconds:

☐☐ , ☐☐ seconds

☐ Successful test
☐ Not successful test ➔ **SKIP TO question 10.3.22. on page 49**

10.3.9. Was the participant likely to fall without the help of the nurse?

☐ Yes
☐ No

10.3.10. **For the "tandem complete for 70 years or more" position**, does the participant manage alone to keep the position?

☐ Yes
☐ No ➔**SKIP TO question 10.3.22. on page 49**

10.3.11. "Tandem complete" for 10 seconds (age 70 or more):

☐☐ , ☐☐ seconds

☐ Successful test
☐ Not successful test ➔ **SKIP TO question 10.3.22. on page 49**
☐ Not applicable

10.3.12. Was the participant likely to fall without the help of the nurse?

- ☐ Yes
- ☐ No

10.3.13. **For the "tandem complete for 69 years or less" position**, does the participant manage alone to keep the position?

- ☐ Yes
- ☐ No ➔**SKIP TO question 10.3.22. on page 49**

10.3.14. "Tandem complete" for 30 seconds (age 69 or less):

☐☐ , ☐☐ seconds

- ☐ Successful test
- ☐ Not successful test ➔ **SKIP TO question 10.3.22. on page 49**
- ☐ Not applicable

10.3.15. Was the participant likely to fall without the help of the nurse?

- ☐ Yes
- ☐ No

10.3.16. **For the « standing on one leg with OPEN eyes » position**, does the participant manage alone to keep the position?

☐ Yes
☐ No ➔ **SKIP TO question 10.3.22. on page 49**

10.3.17. “Standing on one leg with OPEN eyes” for 30 seconds (age 69 or less):

*The participant can try this **once**, before the test begins.*

☐☐ , ☐☐ seconds

☐ Successful test
☐ Not successful test ➔**SKIP TO question 10.3.22. on page 49**

10.3.18. Was the participant likely to fall without the help of the nurse?

☐ Yes
☐ No

10.3.19. **For the « standing with CLOSED eyes on one leg » position**, does the participant manage alone to keep the position?

☐ Yes
☐ No ➔**SKIP TO question 10.3.22.**

10.3.20. "Standing with CLOSED eyes on one leg" for 30 seconds (age 69 or less):

☐☐ , ☐☐ seconds

☐ Successful test
☐ Not successful test

10.3.21. Was the participant likely to fall without the help of the nurse?

☐ Yes
☐ No

10.3.22. Reason for which the balance test was not performed or is not achieved:

☐ Unable to stay standing alone
☐ Lower limb prothesis
☐ Dizziness or lightheadedness
☐ Left foot/leg amputation
☐ Right foot/leg amputation
☐ Left toe(s) amputation
☐ Right toe(s) amputation
☐ Left foot/leg plaster
☐ Right foot/leg plaster
☐ Refusal
☐ Other, specify: ....

10.4. CHAIR RISES

First, the participant will be asked to get up from a chair without using his arms. This test shows whether the participant got muscular strength in the hips and legs ***to stand up and to sit down as fast as possible****. The less muscles are developed, the more the risk of falling is high.*

10.4.1. Did the participant succeed in rising once?

☐ Yes
☐ No ➔**SKIP TO question 10.4.5.**

10.4.2. At the age of 69 years or less, 10 liftings are required:

☐☐ times

☐☐ , ☐☐ seconds (limited time is 60 seconds)

☐ Successful test
☐ Not successful test
☐ Not applicable

10.4.3. At the age of 70 or more, 5 liftings are required:

☐ time

☐☐ , ☐☐ seconds (limited time is 60 seconds)

☐ Successful test
☐ Not successful test
☐ Not applicable

10.4.4. Reason for which the test was interrupted by the study nurse:

- ☐ The participant is tired
- ☐ The participant is out of breath
- ☐ The participant used his hands

10.4.5. Reason for which the balance test was not performed :

- ☐ Unable to stay standing alone
- ☐ Lower limb prothesis
- ☐ Dizziness or lightheadedness
- ☐ Left foot/leg amputation
- ☐ Right foot/leg amputation
- ☐ Left toe(s) amputation
- ☐ Right toe(s) amputation
- ☐ Left foot/leg plaster
- ☐ Right foot/leg plaster
- ☐ Refusal
- ☐ Other, specify: ....

10.5. WALKING SPEED

For each walking test, the participant starts behind the starting line, both feet up on the starting line. The study nurse shows the participant where the 2.44 meter itinerary ends.

10.5.1. Does the participant have one of the following contraindications?

Yes	No	
☐	☐	Symptomatic neuropathy
☐	☐	Symptomatic myelopathy

10.5.2. The study nurse gives the following explanation to the participant:

*"This is our course of walk. If you use a walking aid when you go outside, you can use it for this test. I want you to walk at **your usual rhythm** between the 2 cones. Realise the whole way and stop after the cone. I will walk behind you. We will make this test twice."*

1. ☐☐ , ☐☐ seconds
2. ☐☐ , ☐☐ seconds

10.5.3. Reason for which the walking speed test was not carried out:

- ☐ Unable to stay standing alone
- ☐ Fall during the test
- ☐ Dizziness or lightheadedness
- ☐ Left foot/leg amputation
- ☐ Right foot/leg amputation
- ☐ Left toe(s) amputation
- ☐ Right toe(s) amputation
- ☐ Left foot/leg plaster
- ☐ Right foot/leg plaster
- ☐ Refusal
- ☐ Other, specify: ....

10.6. STEP TEST

10.6.1. Before the beginning of the step test, the study nurse checks if the participant has a contraindication:

	Yes	No
Recent ECG with a significant change: ischemia, myocardial infarction within the 3 last months or another sharp cardiac event.	☐	☐
Angina pectoris	☐	☐
Arrhythmias	☐	☐
History of anevrysm or stenosis	☐	☐
Heart failure	☐	☐
Taking beta blockers	☐	☐
Sharp or chronic problems to walk or to climb up the stairs	☐	☐

➔**If yes, SKIP TO chapter ACCELEROMETER on page 57.**

10.6.2. ID of device: ☐

10.6.3. The test was carried out:

☐ Yes
☐ No ➔**SKIP TO question 10.6.6.**

10.6.4. Number of realised steps per minute:

☐☐ in 1 minute
☐☐ in 2 minutes
☐☐ in 3 minutes
☐☐ in 4 minutes
☐☐ in 5 minutes
☐☐ in 6 minutes
☐☐ in 7 minutes
☐☐ in 8 minutes

10.6.5. At the end of the test, the study nurse observes the pulse for 1 minute on the screen measured by Actiheart, and notes the value after 60 seconds:

☐☐☐ /60 sec.

10.6.6. Reason why the test was not carried out:

- ☐ Dizziness or lightheadedness
- ☐ Nausea that occured during the test
- ☐ Left foot/leg amputation
- ☐ Right foot/leg
- ☐ Left toe(s) amputation
- ☐ Right toe(s) amputation
- ☐ Left foot/leg plaster
- ☐ Right foot/leg plaster
- ☐ Refusal
- ☐ Other, specify: ....

10.7. ACCELEROMETER

The study nurse tells the participant the benefits and gives the instructions how to use the accelerometer.

"The bracelet collects data on your physical activity as well as on your sleep patterns. You will wear it 7 days on the wrist and send it afterwards back to us, using the enclosed envelope."

10.7.1. ID of device: ☐ ☐

10.7.2. The participant agrees to wear the bracelet:

- ☐ Yes
- ☐ No

10.7.3. Reason why the participant prefers not to participate in this test:

- ☐ No time enough
- ☐ Risk of forgetting it
- ☐ Fear of losing it
- ☐ No desire
- ☐ Other, specify: ....

11. END OF NURSE APPOINTMENT

A huge thank you for your time and participation.

Do not hesitate to contact us if you have any questions or concerns.

Time at the end of the appointment: ☐☐ : ☐☐

Appreciation of the participant:

....

....

....
