# Supplementary material for: Challenges and benefits of integrating diverse sampling strategies in the observation of cardiovascular risk factors (ORISCAV-LUX 2) study
Source: BMC Med Res Methodol. 2019 Feb 4;19:27. doi: 10.1186/s12874-019-0669-0 (PMC6360765; doi:10.1186/s12874-019-0669-0)
Supplement: Supplementary file 2 — Questionnaires ORISCAV-LUX 2. (Home-based Questionnaire) English version. (PDF 898 kb) [file 12874_2019_669_MOESM2_ESM.pdf]

YOUR HOME-BASED QUESTIONNAIRE

Dear Madam, Dear Sir,

We thank you in advance for granting us a little of your time.

Preferably choose a **quiet place** in order to answer all questions to the best of your ability.

Tick the box below to give us your consent to this home-based questionnaire participation.

☐	I agree to fill this questionnaire and to bring it with me to my appointment with the study nurse.

1. SOCIAL VARIABLES

1.01. Are you...?

- ☐ A man
- ☐ A woman

1.02. What is your date of birth?

☐☐ / ☐☐ / ☐☐☐☐

Day Month Year

1.03. What is your country of birth?

☐☐☐☐☐☐☐☐☐☐☐☐☐☐☐☐

☐☐☐☐☐☐☐☐☐☐☐☐☐☐☐☐

1.04. What is your current, legal marital status?

- ☐ Married or in a registered life partnership (PACS)
- ☐ Widow(er)
- ☐ Divorced
- ☐ Live in a consensual union
- ☐ Single, never married
- ☐ Separated, but still legally married

1.05. What is your **highest** educational degree **obtained**?

- ☐ No diploma
- ☐ CATP – Certificate of Technical and Professionnal Aptitude
- ☐ CITP - Certificate of Technical and Professional Initiation
- ☐ CCM – Certificate of Manual Capability
- ☐ Diploma for Completion of Secondary Technical Studies
- ☐ Technician diploma
- ☐ Diploma for Completion of Secondary General Studies
- ☐ Bac +2 (BTS)
- ☐ Bac +3 (Bachelors/Degree)
- ☐ Bac +4 (Masters)
- ☐ Bac +5 and more (3rd Cycle, DEA, DESS, MBA, Masters, PhD, etc.)
- ☐ Diploma from a Grande Ecole, an Engineering School
- ☐ Other, please specify : ....

1.06. Which is your **current** activity?

- ☐ Employed
- ☐ In school, university or in training
- ☐ Unemployed or in search of employment
- ☐ Retired or in early retirement
- ☐ At home
- ☐ Invalid
- ☐ On long-term leave (for example: illness)
- ☐ On parental leave
- ☐ Other, please specify : ....

1.07. If you are employed or exercise a profession:

➔ **If you do not work, SKIP TO question 1.10 page 6**

This question refers to paid work. If you have several occupations, consider the ***main one****.*

State the exact designation of your profession*, i.e. do not indicate electrician, but rather electrical contracter; instead of saleswoman indicate saleswoman for shoes.*

What is your current profession? **Describe your main task accurately!**

____

____

Please do not fill in this section!

ISCO-08

☐ ☐

(nn)

1.08. Please use the most precise terms possible to describe the economic activity of your company:

If you do not know this, then mention the name of your company.

____

____

Please do not fill in this section!

NACE Rev.2

☐

(A)

1.09. Do you have a paid job at present?

- ☐ Yes, full time
- ☐ Yes, part time
- ☐ No, ➔ **If no, SKIP to question 1.10.**

If yes, we would like to know the type and amount of physical activity involved in your work.

One single answer is possible.

- ☐ Sedentary occupation: you spend most of your time sitting (such as in an office)
- ☐ Standing occupation: you spend most of your time standing or walking, but your work does not require intense physical effort (e.g., shop assistant, hairdresser, guard)
- ☐ Manual work: this involves some physical effort including handling of heavy objects and use of tools (e.g., plumber, electrician, carpenter)
- ☐ Heavy manual work: this involves very vigorous physical activity including handling very heavy objects (e.g., docker, miner, bricklayer, construction worker)

1.10. How many persons live in your household **(including yourself)**?

☐☐ Adults (over 18 years)

☐☐ Children between 14 years and 18 years

☐☐ Children under 14 years

1.11. What is your monthly or annual net **houshold** income?

If you live with other people at the same postal address, add your net content with that of the other people who live with you and follow a paid-up activity, enclosed aid, pensions, profits from industrial concern and from non-commercial activities, profits from the agriculture.

- ☐ Less than 750 Euros/month (or under 9000 Euros/year)
- ☐ 750 to 1499 Euros/month (or 9000 to 17999 Euros/year)
- ☐ 1500 to 2249 Euros/month (or 18000 à 26999 Euros/year)
- ☐ 2250 to 2999 Euros/month (or 27000 à 35999 Euros/year)
- ☐ 3000 to 4999 Euros/month (or 36000 à 59999 Euros/ year)
- ☐ 5000 to 10000 Euros/month (or 60000 à 119999 Euros/year)
- ☐ More than 10000 Euros/month (or more than 120000 Euros/year)
- ☐ I do not know
- ☐ I do not wish to answer

1.12. To what extent does your current income allow you to provide for your needs?

- ☐ Very difficult
- ☐ Fairly difficult
- ☐ Quite easily
- ☐ Very easily
- ☐ I do not wish to answer

2. QUALITY OF LIFE

The following questions refer to the perception you have of your health.

2.01. What importance do you attach to the following items in order to feel in good health?

	Great importance	Enough importance	Little importance	No importance
Sleep and sufficient rest	☐	☐	☐	☐
Balanced meals	☐	☐	☐	☐
Maintain normal weight	☐	☐	☐	☐
Participate in social and cultural activities	☐	☐	☐	☐
Control stress	☐	☐	☐	☐
Participate in physical activities such as exercise, sports and games	☐	☐	☐	☐
Live in a smoke-free environment	☐	☐	☐	☐

2.02. In general, would you say your health is :

Only one answer is possible.

- ☐ Excellent
- ☐ Very good
- ☐ Good
- ☐ Fair
- ☐ Poor

2.03. **Compared to one year ago,** how would you rate your health in general **now**?

Only one answer is possible.

- ☐ Much better now than one year ago
- ☐ Somewhat better now than one year ago
- ☐ About the same
- ☐ Somewhat worse now than one year ago
- ☐ Much worse than one year ago

2.04. The following items are about activities you might do during a typical day. Does **your health now limit you** in these activities? If so, how much?

Only one answer per line is possible.

	Yes, limited a lot	Yes, limited a little	No, not limited at all
Vigorous activities, such as running, lifting heavy objects, participating in strenuous sports.	☐	☐	☐
Moderate activities, such as moving a table, pushing a vacuum cleaner, bowling.	☐	☐	☐
Lifting or carrying groceries.	☐	☐	☐
Climbing several flights of stairs.	☐	☐	☐
Climbing one flight of stairs.	☐	☐	☐
Bending, kneeling, or stooping.	☐	☐	☐
Walking more than a mile.	☐	☐	☐
Walking several blocks.	☐	☐	☐
Walking one block.	☐	☐	☐
Bathing or dressing yourself.	☐	☐	☐

2.05. During the **past 4 weeks**, have you had any of the following problems with your work or other regular daily activities as a result of your **PHYSICAL health**?

Answer: yes or no at each line.

	Yes	No
Cut down the amount of time you spent on work or other activities.	☐	☐
Accomplished less than you would like.	☐	☐
Were limited in the kind of work or other activities.	☐	☐
Had difficulty performing the work or other activities (for example, it took extra effort).	☐	☐

2.06. During the **past 4 weeks**, have you had any of the following problems with your work or other regular daily activities as a result of any **EMOTIONAL problems** (such as feeling depressed or anxious)?

Answer: yes or no at each line.

	Yes	No
Cut down the amount of time you spent on work or other activities.	☐	☐
Accomplished less than you would like.	☐	☐
Did not do work or other activities as carefully as usual.	☐	☐

2.07. During the **past 4 weeks**, to what extent has your **physical health or emotional problems** interfered with your normal social activities with family, friends, neighbors, or groups? *Only one answer is possible.*

- ☐ Not at all
- ☐ Slightly
- ☐ Moderately
- ☐ Quite a bit
- ☐ Extremely

2.08. How much **bodily pain** have you had during the **past 4 weeks**?
Only one answer possible.

- ☐ None
- ☐ Very mild
- ☐ Mild
- ☐ Moderate
- ☐ Severe
- ☐ Very severe

2.09. During the **past 4 weeks**, how much did **pain** interfere with your normal work (including work outside the home and housework)?

Only one answer is possible.

- ☐ Not at all
- ☐ A little bit
- ☐ Moderately
- ☐ Quite a bit
- ☐ Extremely

The following 9 questions are about how you feel and how things have been with you during the last 4 weeks. For each question, please give the one answer that comes closest to the way you have been feeling.

2.10. How much of the time during the **past 4 weeks**...:

Only one answer per line is possible.

	All of the time	Most of the time	A good bit of the time	Some of the time	A little bit of the time	None of the time
Did you feel full of pep?	☐	☐	☐	☐	☐	☐
Have you been a very nervous person?	☐	☐	☐	☐	☐	☐
Have you felt so down in the dumps that nothing could cheer you up?	☐	☐	☐	☐	☐	☐
Have you felt calm and peaceful?	☐	☐	☐	☐	☐	☐
Did you have a lot of energy?	☐	☐	☐	☐	☐	☐
Have you felt downhearted and blue?	☐	☐	☐	☐	☐	☐
Did you feel worn out?	☐	☐	☐	☐	☐	☐
Have you been a happy person?	☐	☐	☐	☐	☐	☐
Did you feel tired?	☐	☐	☐	☐	☐	☐

2.11. During the **past 4 weeks**, how much of the time has your **physical health or emotional problems** interfered with your social activities (like visiting with friends, relatives, etc.)?

Only one answer is possible.

☐ All of the time ☐ Most of the time ☐ Some of the time ☐ A little of the time ☐ None of the time

2.12. How **TRUE or FALSE is each** of the following statements for you?

Only one answer per line is possible.

	Definitely true	Mostly true	Do not know	Mostly false	Definitely false
I seem to get sick a little easier than other people.	☐	☐	☐	☐	☐
I am as healthy as anybody I know.	☐	☐	☐	☐	☐
I expect my health to get worse.	☐	☐	☐	☐	☐
My health is excellent.	☐	☐	☐	☐	☐

3. MENTAL WELL-BEING

3.01. During the past week:

Only one answer per line is possible.

	Rarely or none of the time (less than 1 day)	Some or a little of the time (1 to 2 days)	Occasionally or a moderate amount of the time (3 to 4 days)	Most or all of the time (5 to 7 days)
I was bothered by things that usually do not bother me.	☐	☐	☐	☐
I did not feel like eating; my appetite was poor.	☐	☐	☐	☐
I felt that I could not shake off the blues even with help from my family or friends.	☐	☐	☐	☐
I felt I was just as good as other people.	☐	☐	☐	☐
I had trouble keeping my mind on what I was doing.	☐	☐	☐	☐
I felt depressed.	☐	☐	☐	☐
I felt that everything I did was an effort.	☐	☐	☐	☐
I felt hopeful about the future.	☐	☐	☐	☐
I thought my life had been a failure.	☐	☐	☐	☐

	Rarely or none of the time (less than 1 day)	Some or a little of the time (1 to 2 days)	Occasionally or a moderate amount of the time (3 to 4 days)	Most or all of the time (5 to 7 days)
I felt fearful.	☐	☐	☐	☐
My sleep was restless.	☐	☐	☐	☐
I was happy.	☐	☐	☐	☐
I talked less than usual.	☐	☐	☐	☐
I felt lonely.	☐	☐	☐	☐
People were unfriendly.	☐	☐	☐	☐
I enjoyed life.	☐	☐	☐	☐
I had crying spells.	☐	☐	☐	☐
I felt sad.	☐	☐	☐	☐
I felt that people dislike me.	☐	☐	☐	☐
I could not get “going”.	☐	☐	☐	☐

4. SOCIAL SUPPORT

4.01. How many persons are so close to you that you can count on them if you have serious personal problems?

- ☐ None
- ☐ 1 or 2
- ☐ 3 to 5
- ☐ 6 or more

4.02. How much concern do people show in what you are doing?

- ☐ A lot
- ☐ Enough
- ☐ Uncertain (this is to say: neither little nor much concern and interest)
- ☐ Little
- ☐ Not at all

4.03. How easy is it to get practical help from neighbours if you should need it?

- ☐ Very easy
- ☐ Easy
- ☐ Possible
- ☐ Difficult
- ☐ Very difficult

5. NUTRITIONAL HABITS

5.01. Are you currently on a diet?

- ☐ Yes
- ☐ No ➔ **If No, SKIP to question 5.02.**

If yes, for what reason? *Several answers possible.*

- ☐ To lower my blood pressure
- ☐ To reduce my cholesterol level
- ☐ To reduce my blood sugar level
- ☐ To lose weight
- ☐ To keep in shape
- ☐ Coeliac disease
- ☐ Gluten/Lactose intolerance
- ☐ Other, please specify: ....

5.02. Over **the past 2 weeks**, how often have you had poor appetite or overeating?

- ☐ Not at all
- ☐ Several days
- ☐ More than half the days
- ☐ Nearly every day

5.03. Do you use spices and/or herbs in your cooking?

For example: basil, mixed herbs (herbes de Provence), coriander, cumin, etc.

- ☐ Yes, always
- ☐ Yes, from time to time
- ☐ No, never
- ☐ I do not know, I do not cook

5.04. Do you use salt and/or stock cubes, Aromat, Maggi **to prepare your meals?**

- ☐ Yes, salt only
- ☐ Yes, salt and other flavorings
- ☐ No, I add nothing
- ☐ I do not know, I do not cook

5.05. Do you put salt in your food **before eating?**

- ☐ Yes, always
- ☐ Yes, from time to time
- ☐ No, never

5.06. What meals or snacks do you eat **every day?**

If you are neither working nor studying, answer only for "Rest days".

	Work days		Rest days	
Breakfast in the morning	☐ Yes	☐ No	☐ Yes	☐ No
Mid-morning snack	☐ Yes	☐ No	☐ Yes	☐ No
Lunch	☐ Yes	☐ No	☐ Yes	☐ No
Afternoon snack	☐ Yes	☐ No	☐ Yes	☐ No
Dinner	☐ Yes	☐ No	☐ Yes	☐ No
After-dinner snack	☐ Yes	☐ No	☐ Yes	☐ No

5.07. In general, how many times per week do you eat **pre-cooked dishes?**

☐☐ time(s)/week

5.08. Do you do the food shopping by yourself?

- ☐ Yes, always
- ☐ Yes, from time to time
- ☐ No, never ➔ **SKIP TO the chapter PHYSICAL ACTIVITY on page 21**

5.09. **When you do the shopping,** do you look at the nutritional information on food packaging?

- ☐ Yes, always
- ☐ Yes, from time to time
- ☐ No, never ➔ **SKIP TO the chapter PHYSICAL ACTIVITY on page 21**

If yes, does it influence your food purchase?

- ☐ Yes, always
- ☐ Yes, from time to time
- ☐ No, never ➔ **SKIP TO the chapter PHYSICAL ACTIVITY on page 21**

If yes, what message on the packaging tends to make you buy a product?

More than one answer possible.

- ☐ "Light" or "For diabetics" headings
- ☐ Calorie content and nutritional values
- ☐ List of ingredients
- ☐ Specific product characteristics (for example rich in Omega 3 or low cholesterol)
- ☐ Other, please specify: ....

6. PHYSICAL ACTIVITY

*The first questions concern the time that you have spent being physically active **during the last week.** This includes the last seven days up to yesterday (including the weekend). Answer all questions, even if you do not think that you are an active person. This includes activities at work or at school, at your home or in your garden, in your travels, or during your moments of relaxation or during sports.*

The time spent doing **INTENSE** physical activities

*This means the activities that require serious physical effort from you, and which made you **breathe with MUCH MORE difficulty than normal**. For example, think of the times when you have carried heavy loads, dug in your garden, gone mountain biking or played soccer.*

Do not include walking!

6.01. During the last week, including the weekend, on how many days did you engage in these types of **intense physical activities** for at least **10 consecutive minutes?**

☐ days / 7 days → **If 0 day, SKIP to question 6.03.**

☐ Do not know → **SKIP to question 6.03.**

6.02. Now think about **1** of these days during last week when you engaged in one or more **intense physical activities**. How much time in total did you spend at it?

☐☐ : ☐☐

Hours : Minutes

☐ Do not know → **SKIP to question 6.03.**

The time spent doing **MODERATE** physical activities

*This means activities which require moderate physical effort from you, and which make you **breathe with a LITTLE MORE difficulty than normal**. Think of times for example when you carried light loads (5-10 Kg), you vacuumed, or you calmly rode a bicycle, or even played volleyball.*

Do not include walking!

6.03. During the last week, including the weekend, on how many days did you engage in this type of **moderate physical activity** for at least **10 consecutive minutes?**

☐ days / 7 days ➔ **If 0 day, SKIP to question 6.05.**

☐ Do not know ➔ **SKIP to question 6.05.**

6.04. Now think about **1** of these days during last week when you engaged in one or more **moderate physical activities**. How much time in total did you spend at it?

☐☐ : ☐☐

Hours : Minutes

☐ Do not know ➔ **SKIP to question 6.05.**

Time spent **WALKING**

We are now going to see how much time you spent walking during last week. This includes walking at work, at school or around the house, walking to move from one place to another, and any other type of walking that you did for relaxation, for sports, for exercise or for pleasure.

6.05. During the last week, including the weekend, on how many days did you **walk** for at least **10 consecutive minutes?**

☐ days / 7 days ➔ **If 0 day, SKIP to question 6.07.**

☐ Do not know ➔ **SKIP to question 6.07.**

6.06. Now, think about **1** of these days during last week when you walked. How much time in total did you **walk?**

☐☐ : ☐☐

Hours : Minutes

☐ Do not know ➔ **SKIP to question 6.07.**

6.07. In general, at what speed did you walk?

- ☐ At a lively pace which made you breathe with much more difficulty than normal.
- ☐ At a moderate pace which made you breathe with a little more difficulty than normal.
- ☐ At a slow pace, which did not involve any change in your breathing.

The time you spent **SITTING**

*This question concerns the time that you spent daily sitting **during the last week** including at work, around the house, at school and during your relaxation time. This does not include days last weekend. It may, for example, include time spent sitting at the office, on transportation, at friends', reading, or sitting or laying down to watch television or use a computer.*

6.08. **During the last 7 days** (without taking the weekend into account), how much time did you spend sitting during a normal day?

Including time spent at home or at your place of work or study.

☐☐ : ☐☐ / day

Hours : Minutes

☐ Do not know

6.09. **During the last 7 days,** how much time did you spend watching television (including DVD) during a ...?

WORKING day	REST day
☐☐ : ☐☐ /day<br>Hours : Minutes	☐☐ : ☐☐ /day<br>Hours : Minutes
☐ Do not know<br>☐ Not applicable	☐ Do not know

6.10. **During the last 7 days,** how much time did you spend at home, in front of a computer including Internet, video game console, visiting websites, checking emails, going on Facebook, Twitter, Netlog; a ...?

WORKING day	REST day
☐☐ : ☐☐ /day<br>Heures : Minutes	☐☐ : ☐☐ /day<br>Heures : Minutes
☐ Do not know<br>☐ Not applicable	☐ Do not know

6.11. Do you normally do sport?

☐ Yes
☐ No ➔ **SKIP TO question 6.13.**

6.12. Which kind of sport do you do?

☐☐☐☐☐☐☐☐☐☐☐☐☐☐☐☐
☐☐☐☐☐☐☐☐☐☐☐☐☐☐☐☐

➔ **SKIP TO chapter SLEEP QUALITY on page 26 ...**

6.13. If you do not normally do any kind of sport, what are the reasons?

More than one response possible.

- ☐ I am not fond of sport
- ☐ Because of health problems
- ☐ For professional reasons
- ☐ Due to age
- ☐ For lack of time
- ☐ Other, please specify: ....

7. SLEEP QUALITY

*The following questions relate to your usual sleep habits during the past month only. Your answers should indicate the most accurate reply for the majority of days and nights **in the past month**.*

Please answer all questions.

7.01. Do you have a job or a professional occupation with different cycles or working timetables (e.g., confectioner, night-shift nurse, truck driver, cashier in a hypermarket, etc.)?

☐ Yes

☐ No

7.02. During the **past month**, what time have you usually gone to bed **at night?**

Bed time: ☐☐ : ☐☐

Hours : Minutes

7.03. During the **past month**, how long (in minutes) has it usually taken you to fall asleep each night?

Number of minutes: ☐☐

7.04. During the **past month**, what time have you usually gotten up in the morning?

Getting up time: ☐☐ : ☐☐

Hours : Minutes

7.05. During the **past month**, how many hours of actual sleep did you get at night?

This number may be different from the number of hours you spent in bed.

Hours of sleep per night: ☐☐ : ☐☐

Hours : Minutes

For each of the remaining questions, check the one best response. Please answer all the questions.

7.06. During the **past month**, how often have you had trouble sleeping because you...

	Not during the past month	Less than once a week	Once or twice a week	Three or more times a week
Cannot get to sleep within 30 minutes.	☐	☐	☐	☐
Wake up in the middle of the night or early morning.	☐	☐	☐	☐
Have to get up to use the bathroom.	☐	☐	☐	☐
Cannot breath comfortably.	☐	☐	☐	☐
Cough or snore loudly.	☐	☐	☐	☐
Feel too cold.	☐	☐	☐	☐
Feel too hot.	☐	☐	☐	☐
Had bad dreams.	☐	☐	☐	☐
Have pain.	☐	☐	☐	☐
Other reasons, please describe:	☐	☐	☐	☐

7.07. During the **past month**, how would you rate your sleep quality overall?

- ☐ Very good
- ☐ Fairly good
- ☐ Fairly bad
- ☐ Very bad

7.08. During the **past month**, how often have you taken medecine to help you sleep (prescribed or “over the counter”)?

- ☐ Not during the past month
- ☐ Less than once a week
- ☐ Once or twice a week
- ☐ Three or more times a week

7.09. During the **past month**, how often have you had trouble staying awake while driving, eating meals or engaging on social activity?

- ☐ Not during the past month
- ☐ Less than once a week
- ☐ Once or twice a week
- ☐ Three or more times a week

7.10. During the **past month**, how much of a problem has it been for you to keep up enough enthusiasm to get things done?

- ☐ No problem at all
- ☐ Only a very slight problem
- ☐ Somewhat of a problem
- ☐ A very big problem

7.11. Do you have a bed partner or room mate?

- ☐ No bed partner or room mate. → **SKIP TO chapter INTAKE OF VITAMIN D on page 30**
- ☐ Partner/room mate in other room.
- ☐ Partner in same room, but not same bed.
- ☐ Partner in same bed.

7.12. If you have a room mate or bed partner, ask him/her how often **in the past month** you have had:

	Not during the past month	Less than once a week	Once or twice a week	Three or more times a week
Loud snoring	☐	☐	☐	☐
Long pauses between breaths while asleep	☐	☐	☐	☐
Legs twitching or jerking while you sleep	☐	☐	☐	☐
Episodes of disorientation or confusion during sleep	☐	☐	☐	☐
Other restlessness while you sleep, please describe:	☐	☐	☐	☐

8. INTAKE OF VITAMIN D

8.01. Do you go under the solarium?

- ☐ Never
- ☐ Less than 10 sessions per year
- ☐ Between 10 and 50 sessions per year
- ☐ More than 50 sessions per year

8.02. Do you go out in the sun, when it is nice? (at least uncovered arms and legs)?

	Never	Rarely	Sometimes	Often	Always
During the year	☐	☐	☐	☐	☐
When you are on holiday (abroad)	☐	☐	☐	☐	☐

8.03. When you go out into the sun, do you use some sun-protection cream?

- ☐ Never ➔ **SKIP TO chapter THYROID HEALTH on page 31**
- ☐ Rarely
- ☐ Sometimes
- ☐ Often
- ☐ Always

8.04. What is its indication of protection UV?

- ☐ 4 to 12
- ☐ 15 to 25
- ☐ 30 to 50
- ☐ More than 50
- ☐ I do not know

9. THYROID HEALTH

9.01. Have you ever had any thyroid problems diagnosed by a medical doctor?

- ☐ Yes
- ☐ No ➔ **SKIP TO chapter HEALTH OF DIGESTIVE PROCESS on page 32**

9.02. Have you ever been operated for a thyroid problem?

- ☐ Yes
- ☐ No ➔ **SKIP TO chapter HEALTH OF DIGESTIVE PROCESS on page 32**

If yes, have you had any surgery in the **last 12 months?**

- ☐ Yes
- ☐ No

Do you know why you have been operated?

....

- ☐ I do not know

10. HEALTH OF DIGESTIVE PROCESS

Constipation is hard, dry, lumpy stools that are difficult or painful to pass and which may be accompanied by bloating and discomfort.

10.1. What is the frequency of bowel movements?

- ☐ Each day
- ☐ Every 2 days
- ☐ 2 times per week
- ☐ Once per week
- ☐ Less than once per week
- ☐ Less than once per month

10.2. At what frequency do you have difficulty in having a bowel movement (painful evacuation effort)?

- ☐ Never
- ☐ Rarely

➡ **SKIP TO chapter POLLUTION on page 34**

- ☐ Sometimes
- ☐ Usually
- ☐ Always

10.3. Do you use any type of assistance to have a bowel movement?

- ☐ Without assistance
- ☐ Stimulative laxatives
- ☐ Digital assistance or enema

10.4. How much time do you spend on lavatory per attempt?

- ☐ Less than 5 minutes
- ☐ Between 5 and 10 minutes
- ☐ Between 10 and 20 minutes
- ☐ Between 20 and 30 minutes
- ☐ More than 30 minutes

10.5. How many unsuccessful attempts for evacuation do you have per 24 hours?

- ☐ 0 times/day
- ☐ 1 to 3 times/day
- ☐ 4 to 6 times/day
- ☐ 7 to 9 times/day
- ☐ More than 9 times/day

10.6. At what frequency do you note a feeling of incomplete evacuation?

- ☐ Never
- ☐ Rarely
- ☐ Sometimes
- ☐ Usually
- ☐ Always

10.7. How often do you have abdominal pain?

- ☐ Never
- ☐ Rarely
- ☐ Sometimes
- ☐ Usually
- ☐ Always

10.8. What is the duration of constipation?

- ☐ Less than 1 year
- ☐ Between 1 and 5 years
- ☐ Between 5 and 10 years
- ☐ Between 10 and 20 years
- ☐ More than 20 years

11. POLLUTION

WORKING AND LIVING CONDITIONS

The next set of questions is on your working and living conditions. This information will allow us to see if the environmental living and working conditions have an impact on health.

11.01. When has the building you live in been built?

- ☐ Less than 10 years ago
- ☐ Between 10 years and 30 years ago
- ☐ Over 30 years ago
- ☐ I do not know

If the building is **over 30 years** old, can you state exactly how old it is?

☐☐☐ years and ☐☐ months

☐ I do not know

11.02. For how many years do you live in the current accommodation?

☐☐ years

11.03. Do you apply external treatments against fleas and ticks to your domestic animals?

- ☐ Yes
- ☐ No
- ☐ I do not have any domestic animals

11.04. Do you use pesticides (for example herbicides, insecticides, fungicides, etc.) inside your home?

- ☐ Yes
- ☐ No ➔ **SKIP TO question 11.05.**

If yes, for which purpose are you using them? *More than one response possible.*

- ☐ To treat my plants
- ☐ Against flies, mosquitoes, spiders, cockroaches, moths, etc.
- ☐ Others, please specify: ....

11.05. Do you use pesticides in your garden?

- ☐ Yes
- ☐ No
- ☐ I do not have a garden

11.06. How far do you live from a heavy traffic road?

- ☐ Less than 100 m
- ☐ More than 100 m and less than 500 m
- ☐ More than 500 m ➔ **SKIP TO the instruction at the end of this page**

11.07. What kind of road is it?

- ☐ Motorway / Highway
- ☐ Main road in town or urban area
- ☐ Main road outside town or urban area
- ☐ Other, please specify: ....

INSTRUCTION:
If you do not have a job or any professional occupation ➔ **SKIP TO question 11.14. on page 38**

11.08. If you have a job or any professional occupation, what is your present working address (street and city)?

Street: ....

City: ....

11.09. Is it part of your job to commute regularly or daily to another place to do there your work (for example a nurse aide in home care, a taxi driver, a bricklayer at different construction sites, etc.)?

- ☐ Yes
- ☐ No

11.10. If you have a job or any professional occupation, how far do you work from a heavy traffic road?

- ☐ Less than 100 m
- ☐ More than 100 m and less than 500 m
- ☐ More than 500 m ➔ **SKIP TO question 11.12.**

11.11. What kind of road is it?

- ☐ Motorway / Highway
- ☐ Main road in town or urban area
- ☐ Main road outside town or urban area
- ☐ Other, please specify: ....

11.12. How do you travel to work?

If you use more than one means of transportation, please indicate the most frequent or the one you spend most time in.

Example1: 10 minutes by car, 60 minutes by bus => check the bus
Example2: 30 minutes by car and 30 minutes by train => write down in "Other" "car + train".

- ☐ Car
- ☐ Bus
- ☐ Train
- ☐ Bike
- ☐ On foot
- ☐ Working address and residential address the same
- ☐ Other, please specify: ....

11.13. How much time do you need **to travel to work and back from work** every day?

☐☐ : ☐☐ / day
Hours : Minutes

11.14. Have you worked, in the **last 12 months**, in one or more of the following sectors?

1. Public construction and open air
2. Industry
3. Transportation
4. Catering industry and entertainment

For the unemployed persons, the pensioners and the persons in professional training: note please the last domain.

☐ Yes
☐ No ➔ **If no, SKIP TO chapter TOBACCO on page 40**

If yes, more than one response is possible:

1. Public construction and open air:

☐ Road maintenance (work with bitumen, asphalt, etc.)
☐ City maintenance
☐ Maintenance of green spaces
☐ Landscape gardener
☐ Lumberjack, Woodcutter
☐ Agriculture, viticulture
☐ Building sites
☐ Traffic and parking checks, inspection
☐ Terraces / cafés close to road
☐ Other, please specify: ....

2. **Industry:**

- ☐ Mines
- ☐ Metallurgy: iron, steel, aluminium
- ☐ Energie: fuel smelter or recycling (gasoline, diesel, etc.)
- ☐ Car: car construction, tire production, car repair shop, vehicle inspection
- ☐ Chemical / plastics
- ☐ Incinerator: rubbish, waste, wood, etc.
- ☐ Other, please specify: ....

3. **Transportation:**

- ☐ Truck driver
- ☐ Bus driver or taxi driver
- ☐ Salesman in town, deliverer
- ☐ Other, please specify: ....

4. **Catering industry, entertainment:**

- ☐ Cook
- ☐ Waiter, barman
- ☐ Swimming pool: maintenance, instructor
- ☐ Other, please specify: ....

12. TOBACCO

12.01. Do you currently smoke (all kind of tobacco including)?

- ☐ Yes, every day, at least 1 cigarette / day ➔ **SKIP TO question 12.05.**
- ☐ Yes, from time to time, less than 1 cigarette / day ➔ **SKIP TO question 12.05.**
- ☐ Yes, but only electronic cigarettes
- ☐ No

Have you smoked in the past?

- ☐ Yes ➔ **SKIP TO question 12.02.**
- ☐ No ➔ **SKIP TO chapter ALCOHOL on page 44**

FOR PAST SMOKERS

12.02. If you no longer smoke, for how many years did you smoke?

☐☐ years

12.03. If you no longer smoke, how many cigarettes did you smoke per day on average?

☐☐ cigarette(s)/ day

12.04. If you no longer smoke, before how many years did you stop?

☐☐ years ➔ **SKIP TO question 12.14 on page 43**

FOR REGULAR SMOKERS

12.05. How soon after you wake up do you smoke your first cigarette?

- ☐ Within 5 minutes
- ☐ 6 to 30 minutes
- ☐ 31 to 60 minutes
- ☐ After 60 minutes

12.06. Do you find it difficult to refrain from smoking in places where it is forbidden (e.g., at the library, cinema)?

- ☐ Yes
- ☐ No

12.07. Which cigarette would you hate most to give up?

- ☐ The first one in the morning
- ☐ All others

12.08. How many cigarettes per day do you smoke?

- ☐ 10 or less
- ☐ 11 to 20
- ☐ 21 to 30
- ☐ 31 or more

12.09. Do you smoke more frequently during the first hours of waking up than during the rest of the day?

- ☐ Yes
- ☐ No

12.10. Do you smoke if you are so ill that you are in bed most of the day?

- ☐ Yes

☐ No

12.11. Have you already considered giving up tobacco?

☐ Yes
☐ No → **SKIP TO question 12.13.**

If yes, how many times?

☐ Once
☐ Twice
☐ 3 times
☐ If more, specify: ☐☐ times

12.12. During your **last attempt** how long did you go without smoking?

☐☐ days
☐☐ weeks
☐☐ months
☐☐ years

12.13. Are you concerned about the harmful effects that the tobacco that you smoke may have on your own health?

☐ Very concerned
☐ Fairly concerned
☐ Slightly concerned
☐ Not at all concerned

12.14. What was the main reason that you started smoking?

☐ I wanted to smoke
☐ Advertising
☐ My spouse smokes
☐ My friends
☐ My parents

- [ ] Other, please specify: ………………………………………………………..........

12.15. What is (or will be) the main reason leading you (or that will lead you) to stop smoking?

- [ ] Fear of falling ill
- [ ] The birth of a child, pregnancy
- [ ] The price of cigarettes
- [ ] Getting sick or a health problem
- [ ] Anti-smoking campaign
- [ ] Family
- [ ] People around you besides family, friends, colleagues
- [ ] Awareness of the consequences of tobacco on health
- [ ] General environment with regard to tobacco, social image
- [ ] No particular reason
- [ ] Do not know
- [ ] Other, please specify: ……………………………………………………........

13. ALCOHOL

13.01. Have you ever consumed alcohol, apart from a few sips or trials?

- ☐ Yes
- ☐ No, not in my whole life ➔ **SKIP TO page 49**

13.02. How **old** were you when you have consumed an alcoholic drink **for the first time?**

☐☐ years

Please continue on the following page...

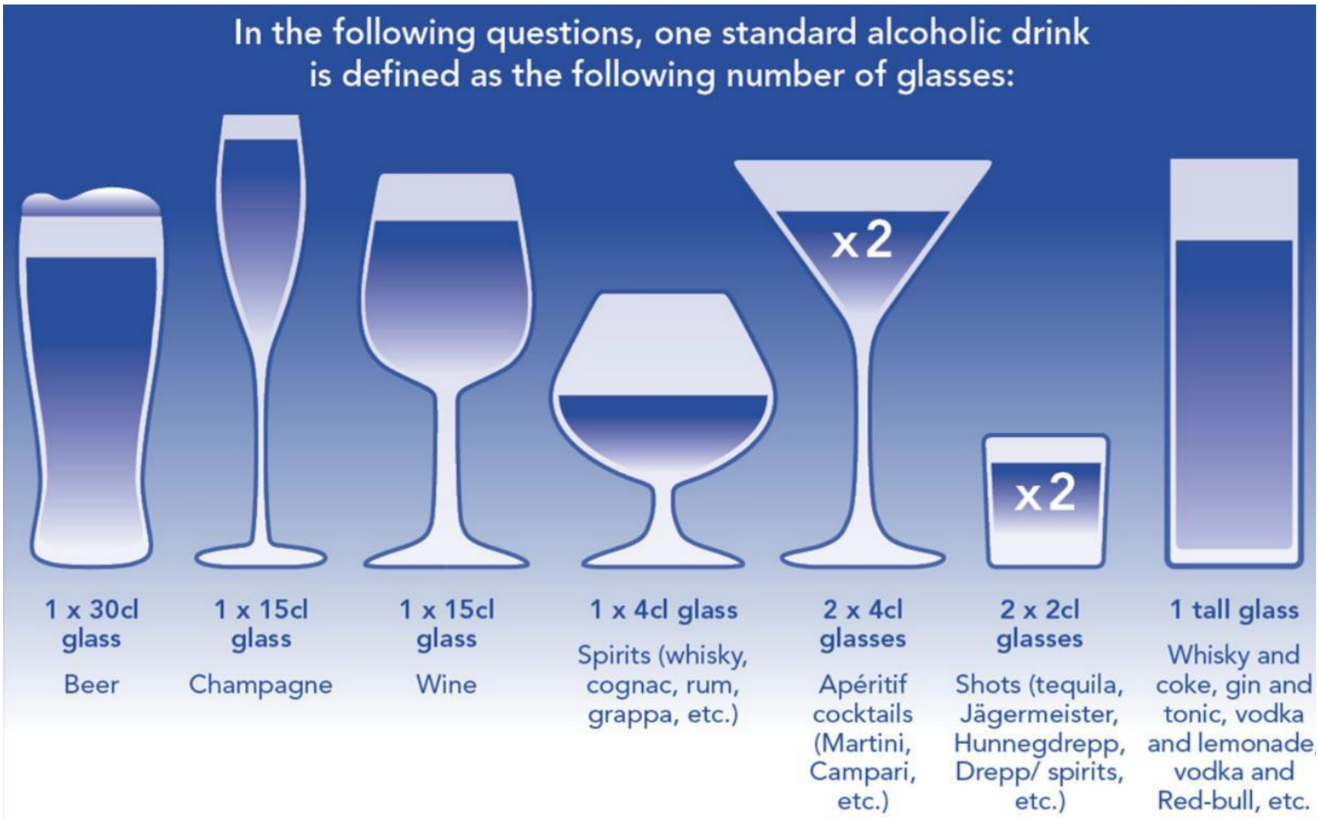

13.03. During the last 12 months, how often have you had a drink containing alcohol?

- ☐ Never ➔ **SKIP TO page 49**
- ☐ Monthly or less
- ☐ 2 to 4 times a month
- ☐ 2 to 3 times a week
- ☐ 4 or more times a week

13.04. How many drinks containing alcohol do you have on a typical day when you are drinking?

- ☐ 1 or 2
- ☐ 3 or 4
- ☐ 5 or 6
- ☐ 7 to 9
- ☐ 10 or more

13.05. How often do you have five or more drinks on one occasion?

- ☐ Never
- ☐ Less than monthly
- ☐ Monthly
- ☐ Weekly
- ☐ Daily or almost daily

13.06. How often during the last year have you found that you were not able to stop drinking once you had started?

- ☐ Never
- ☐ Less than monthly
- ☐ Monthly
- ☐ Weekly
- ☐ Daily or almost daily

13.07. How often during the last year have you failed to do what was normally expected of you because of drinking?

- ☐ Never
- ☐ Less than monthly
- ☐ Monthly
- ☐ Weekly
- ☐ Daily or almost daily

13.08. How often during the last year have you needed a first drink in the morning to get yourself going after a heavy drinking session?

- ☐ Never
- ☐ Less than monthly
- ☐ Monthly
- ☐ Weekly
- ☐ Daily or almost daily

13.09. How often during the last year have you had a feeling of guilt or remorse after drinking?

- ☐ Never
- ☐ Less than monthly
- ☐ Monthly
- ☐ Weekly
- ☐ Daily or almost daily

13.10. How often during the last year have you been unable to remember what happened the night before because of your drinking?

- ☐ Never
- ☐ Less than monthly
- ☐ Monthly
- ☐ Weekly
- ☐ Daily or almost daily

13.11. Have you or someone else been injured because of your drinking?

- ☐ No
- ☐ Yes, but not in the last year
- ☐ Yes, during the last year

13.12. Has a relative, friend, doctor, or other health care worker been concerned about your drinking or suggested you cut down?

- ☐ No
- ☐ Yes, but not in the last year
- ☐ Yes, during the last year

You have reached the END of the questionnaire.

We thank you for your involvement.
